# Genetic diversity of armored scales (Hemiptera: Diaspididae) and soft scales (Hemiptera: Coccidae) in Chile

**DOI:** 10.1038/s41598-017-01997-6

**Published:** 2017-05-17

**Authors:** P. Amouroux, D. Crochard, J.-F. Germain, M. Correa, J. Ampuero, G. Groussier, P. Kreiter, T. Malausa, T. Zaviezo

**Affiliations:** 1 Departamento de Fruticultura y Enología, Facultad de Agronomía e Ingeniería Forestal, Pontificia Universidad Católica de Chile, Santiago, Chile; 2 INRA, Université Côte d’Azur, CNRS, UMR 1355-7254 Institut Sophia Agrobiotech, 06900 Sophia, Antipolis France; 3 ANSES, Laboratoire de la Santé des Végétaux, unité Entomologie et Plantes Invasives, CBGP, 755 avenue du Campus Agropolis, 34988 Montferrier-sur-Lez, France; 4 Xilema-ANASAC Control Biológico, San Pedro, Quillota Chile

## Abstract

Scale insects (Sternorrhyncha: Coccoidea) are one of the most invasive and agriculturally damaging insect groups. Their management and the development of new control methods are currently jeopardized by the scarcity of identification data, in particular in regions where no large survey coupling morphological and DNA analyses have been performed. In this study, we sampled 116 populations of armored scales (Hemiptera: Diaspididae) and 112 populations of soft scales (Hemiptera: Coccidae) in Chile, over a latitudinal gradient ranging from 18°S to 41°S, on fruit crops, ornamental plants and trees. We sequenced the COI and 28S genes in each population. In total, 19 Diaspididae species and 11 Coccidae species were identified morphologically. From the 63 COI haplotypes and the 54 28S haplotypes uncovered, and using several DNA data analysis methods (Automatic Barcode Gap Discovery, K2P distance, NJ trees), up to 36 genetic clusters were detected. Morphological and DNA data were congruent, except for three species (*Aspidiotus nerii*, *Hemiberlesia rapax* and *Coccus hesperidum*) in which DNA data revealed highly differentiated lineages. More than 50% of the haplotypes obtained had no high-scoring matches with any of the sequences in the GenBank database. This study provides 63 COI and 54 28S barcode sequences for the identification of Coccoidea from Chile.

## Introduction

Scale insects (Hemiptera: Coccoidea) are key pests on crops and ornamental plants worldwide. The three most important families in terms of economic damage and number of genera are Diaspididae (419 genera), Pseudococcidae (272 genera) and Coccidae (170 genera)^1^. Scale insects can cause economic damage directly, through sap sucking, the injection of toxins and the transmission of viruses that weaken the plant, lowering fruit quality and yield. Mealybugs (Pseudococcidae) and soft scales (Coccidae) may also cause indirect damage, by excreting honeydew onto the plant surface, thereby favoring the development of sooty mold fungi. Scale insects are also invasive pests^2^ for which quarantine is required in several markets, leading to the rejection of products during international trade. As a result, active inspections for these insects are carried out by national phytosanitary services, and their presence is monitored by most farm advisors. One of the principal problems in scale insect management concerns the difficulty of their identification. Morphological identification is possible but difficult: it requires high level of expertise and the examination of adult females, which are not always found in the field or in the commercial products. Moreover, even when adult females are examined, it is sometimes difficult to distinguish between very similar species^3, 4^. These difficulties have resulted in an overall lack of knowledge for fine-scale taxonomic identification^5, 6^ and, probably, widespread errors in management, with the implementation of control methods that are inappropriate for the species actually present in the target area^7^. In this context, the use of DNA barcoding has gradually increased over the last decade, as a means of disentangling complexes of cryptic species and surveying the biodiversity occurring in various regions of the world^8, 9^. Studies have been performed on the Diaspididae^10–12^, Pseudococcidae^12–16^ and Coccidae^3, 17^. Phylogenetic studies have also provided useful information for the barcoding of scale insects^18, 19^. However, most of these studies have focused on the insects of North America, Asia and Europe. Morphological studies have been performed on scale insects from South America^20–22^, but the marked lack of genetic information for scale insects from this continent is problematic for the efficient management of these pest species in this region. Previous DNA barcoding studies on scale insects have focused on the Pseudococcidae in Chile^14, 23^ and Brazil^24^. The Diaspididae and Coccidae families remain poorly characterized and underexplored.

We present here a survey of the Diaspididae and Coccidae over a latitudinal gradient in Chile, with the aim of providing a comprehensive genetic barcoding database. We collected scale insects from various fruit crops, ornamental plants and trees and shrubs growing next to crops and sequenced their COI and 28S genes. Our objectives were: (i) to survey the biodiversity of scales, (ii) to associate barcode sequences to each of the Chilean species identified on the basis of morphological characters; (iii) to explore the diversity of DNA sequences as a means of unraveling possible complexes of cryptic species. Chile is a particularly interesting location for studies of diversity via DNA barcoding. Its unique geography resembles that of an island and its environmental features create highly diverse agroecosystems and structured insect populations^23^. These conditions favor the establishment of new invading pests and the colonization of crops by native species, as observed for mealybugs infesting vineyards^14, 25^. Survey data are also of direct interest to stakeholders in Chile, such as the Chilean National Agricultural and Livestock Service (*Servicio Agrícola y Ganadero*), which carries out phytosanitary checks on exported products and prevents the introduction of new pests that might seriously damage Chilean agriculture.

## Results

### Coccidae

Eight Coccidae species were identified on the basis of morphology: *Ceroplastes sinensis* Del Guercio, *Coccus hesperidum* Linnaeus, *Parasaissetia nigra* (Nietner), *Parthenolecanium corni* (Bouché), *Protopulvinaria pyriformis* Cockerell, *Pulvinariella mesembryanthemi* (Vallot), *Saissetia coffeae* (Walker), *Saissetia oleae* (Olivier). Three other members of the Coccidae could not be identified to species level: two from the genus *Ceroplastes* (referred to as *Ceroplastes* sp. I and *Ceroplastes* sp. II in this article), and another that could not be identified beyond family level (referred to as unidentified Coccidae). For these three species, only immature stages were collected, precluding morphological identification to species level.

**Figure 1:**
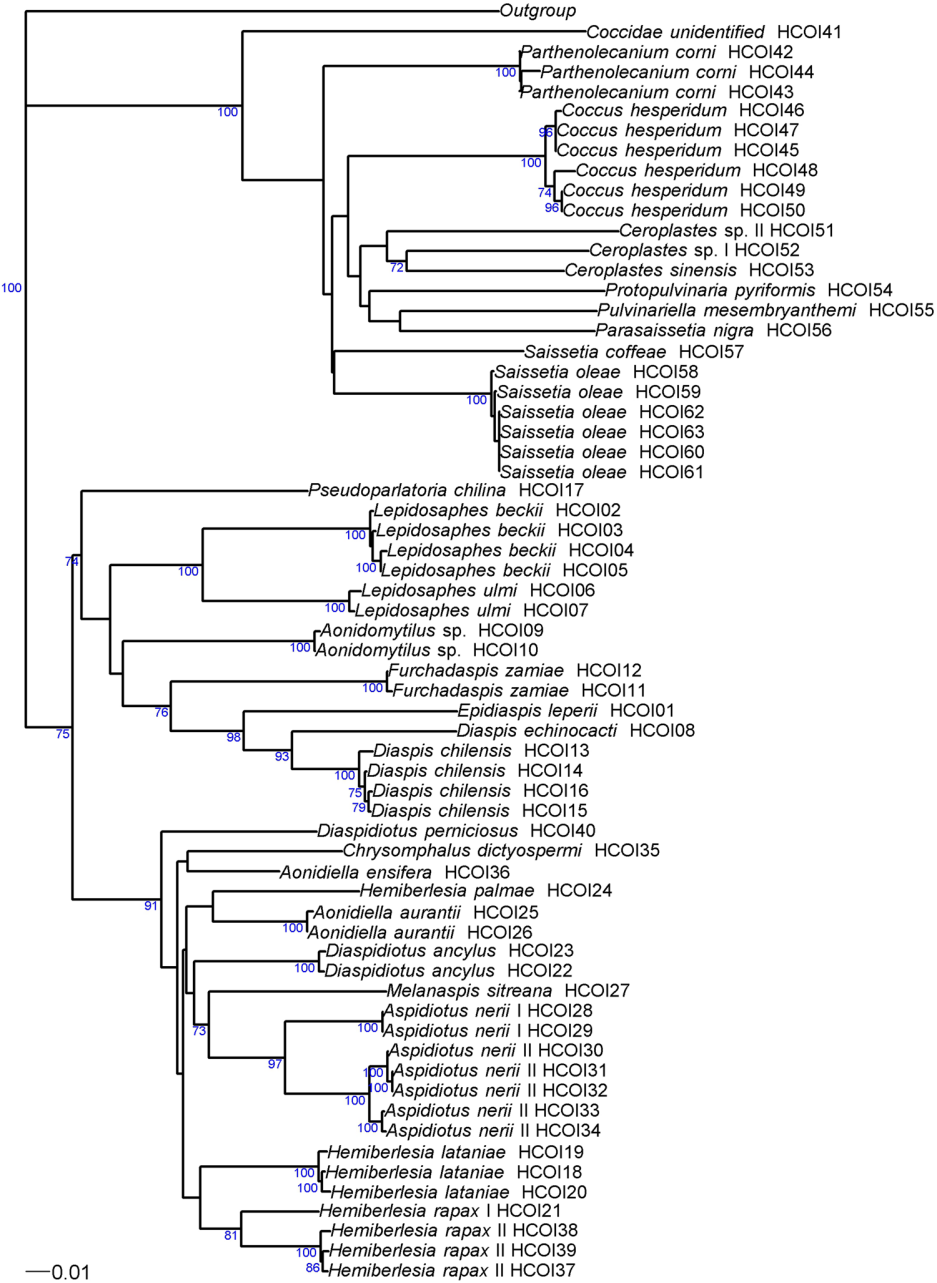
Phylogenetic relationships between 63 haplotypes of Coccidae and Diaspididae based on COI sequences analyzed by neighbor-joining, with the K2P distance model, and *Pseudococcus viburni* (Hemiptera: Pseudococcidae) (GenBank accession: KJ530624) as the outgroup. Bootstrap values below 70% are not shown.

**Figure 2:**
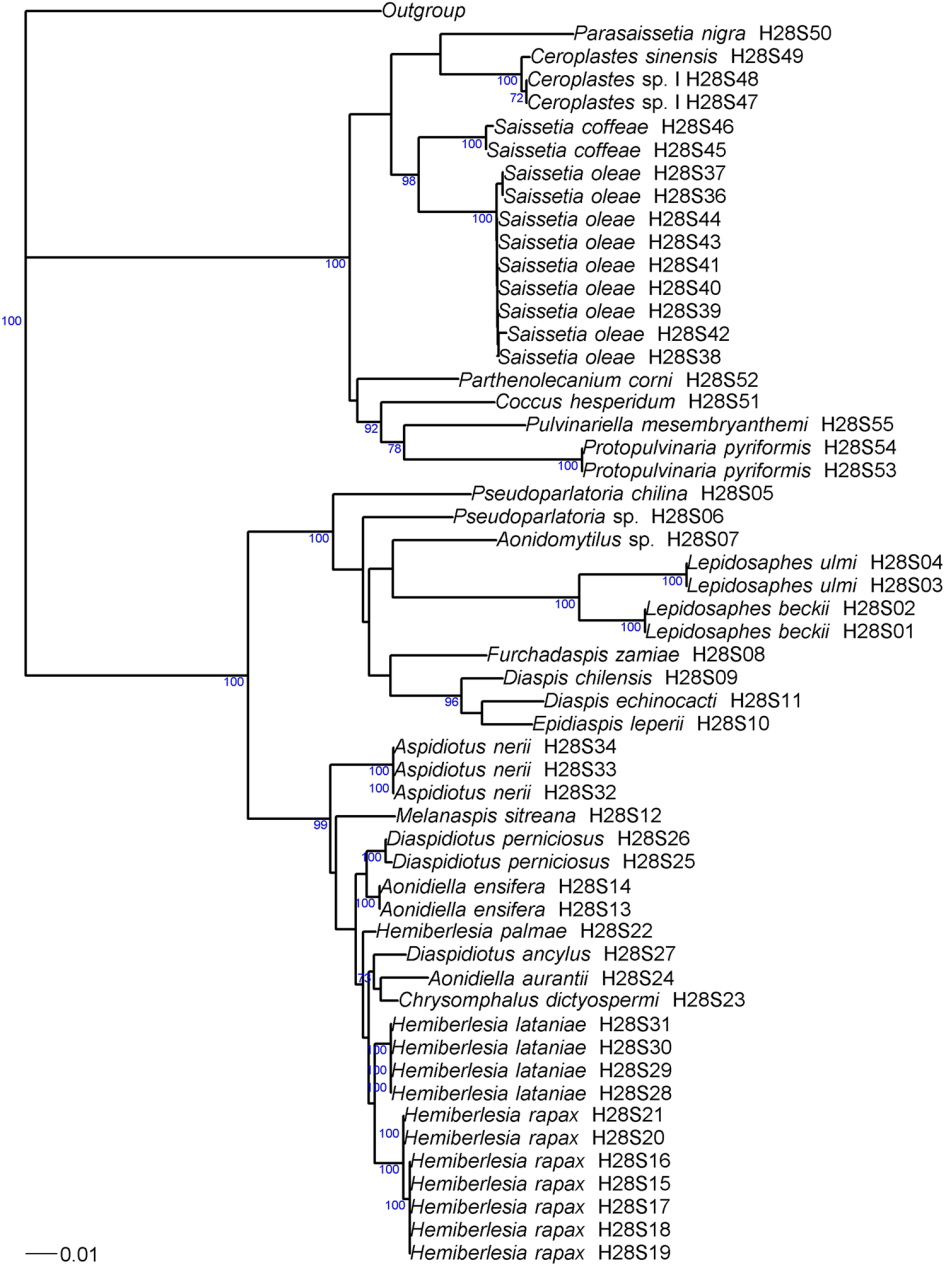
Phylogenetic relationships between 54 haplotypes of Coccidae and Diaspididae based on 28S sequences analyzed by neighbor-joining, with the K2P distance model, and *Pseudococcus viburni* (Hemiptera: Pseudococcidae) (GenBank accession: KU499443) as the outgroup. Bootstrap values below 70% are not shown.

In total, 293 individuals from the Coccidae were processed for molecular analyses; 194 (66%) COI sequences and 220 (75%) 28S sequences were obtained. At least one gene was successfully sequenced in 253 individuals and both genes were sequenced in 161 individuals. For COI, we identified 23 different haplotypes and sequence length was 649 bp. All COI sequences had a bias toward low GC content (A = 40.6%, T = 37.6%, C = 14.8% and G = 7.0%), with a mean GC content of about 21.8% (range: 19.4–24.8%). For 28 S, 20 different haplotypes were identified and sequence length ranged from 788 bp in *P*. *pyriformis* to 818 bp in *P*. *corni*. No bias was observed for 28S sequences. Neighbor-joining trees were plotted for COI and 28S (see Figs 1 and 2, respectively, also including the Diaspididae found). The initial ABGD partition delineated 12 groups within the Coccidae while the recursive partition delineated 13 groups. The difference in the number of clusters with the morphological identification is due to the splitting of *Coccus hesperidum* into two and three groups, respectively.

**Figure 3:**
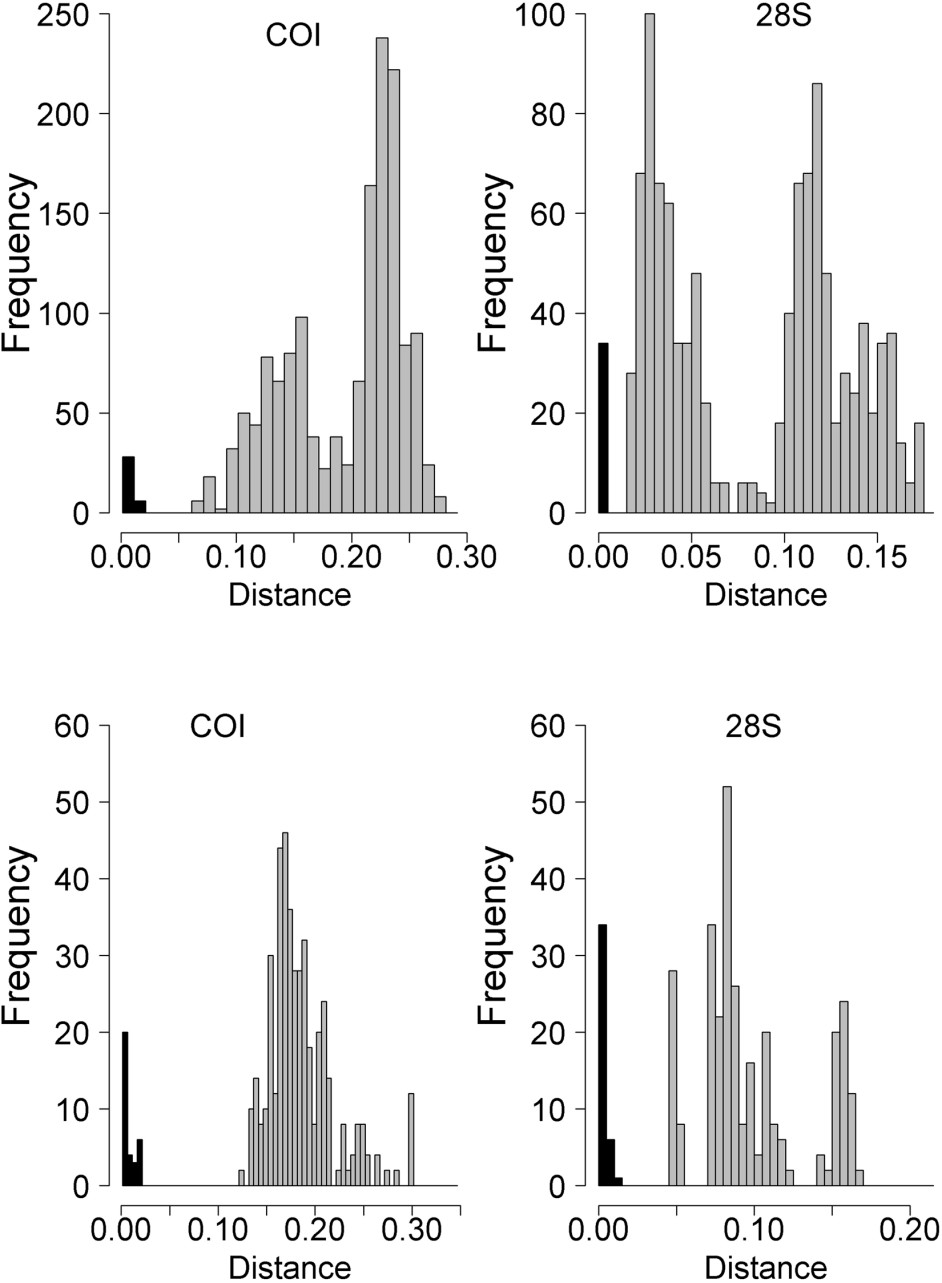
Intraspecific (black) and interspecific (gray) distances for Coccidae (**A**) and Diaspididae (**B**), for COI (left) and 28S (right). Distances were calculated with the Kimura two-parameter nucleotide substitution model.

Figure 3A shows the distribution of intraspecific (black) and interspecific (gray) pairwise distances according to the K2P nucleotide substitution model. We analyzed intra- and interspecific K2P distances with 11 species for COI and 8 species for 28S. The overall difference in the number of clusters between the two genes is due to (i) the merging of *Ceroplastes sinensis* and *Ceroplastes* sp I into a single cluster for 28S, and (ii) the unavailability of 28S sequences for *Ceroplastes sp*. II and the unidentified Coccidae. A barcode gap was observed for intra- and interspecific distances, for both genes. The intraspecific distances were between 0% and 2.0% for COI and between 0% and 0.6% for 28S. The interspecific distances ranged from 12.5% to 30.1% for COI and from 0.9% to 16.7% for 28S. The minimum interspecific distance for 28S (0.9%) was found between *Ceroplastes sp*. I and *C*. *sinensis*.

Species identifications corresponded to the scientific names of the best GenBank hits, with a percentage similarity exceeding 99.6% for the COI haplotypes of *Parasaissetia nigra*, *Parthenolecanium corni*, *Protopulvinaria pyriformis* and one of six haplotypes of *Coccus hesperidum* (Supplementary Data 2). Two haplotypes and their best GenBank hits were mismatched, with a percentage of similarity exceeding this threshold: two of the six COI haplotypes of *Coccus hesperidum* were matched with *Unaspis yanonensis* (Kuwana) (Hemiptera: Diaspididae) (GenBank accession: KP981079). For 28S, species identifications corresponded to the scientific names of the best GenBank hits, also with a percentage similarity exceeding 99.6% for the haplotypes of *Parasaissetia nigra*, *Parthenolecanium corni*, *Protopulvinaria pyriformis* and *Saissetia coffeae*. No mismatches with a percentage similarity exceeding this threshold were observed. The best GenBank hits for the nine 28S haplotypes of *Saissetia oleae* corresponded to *Saissetia miranda* (Cockerell & Parrott), with a percentage similarity of 98.7% to 99.4%. For the 28S haplotypes of *Ceroplastes* sp. I and *C*. *sinensis*, the best GenBank hit had a percentage similarity below 94%. The results of Blastn queries for each haplotype are provided in Supplementary Data 2.

The most widespread Coccidae were *Coccus hesperidum*, which was sampled from 20°S to 39°S (at less than 200 m above sea level), and *Saissetia oleae*, which was sampled from 19°S to 36°S (at elevations of up to 1,000 m above sea level) (Table 1). By contrast, *Saissetia coffeae* was found only in the northernmost regions of Chile (from 18°S to 29°S, at up to 830 m above sea level) and *Pulvinariella mesembryanthemi* was found only in the southern regions of Chile (from 39°S to 40°S). *Parasaissetia nigra* was found at only one site (30°S). The Coccidae with the largest numbers of host plants were *S*. *oleae* and *C*. *hesperidum*, which were collected on 26 host plants (from ten orders) and 12 host plants (from six orders), respectively (Supplementary Data 1 and 3). *Ceroplastes* sp. I was found on *Vaccinium corymbosum* (Ericaceae) and *Berberis* sp. (Berberidaceae), whereas *Ceroplastes* sp. II was collected from *Schinus molle* (Anacardiaceae), a tree endemic to South America. The complete list of host plants for each species is provided in Supplementary Data 1 and 3 (by host plant family and order).

**Table 1:** List, by family and alphabetical order, of the 21 species of Diaspididae and 13 species of Coccidae surveyed, their distribution (from North to South) and the number of individuals sequenced (Nb).

	Administrative Region	XV	I	III	IV	V	Met	VI	VII	VIII	XIV	X	Nb
	Latitude (S)	18°	19°	28°	30°	32°	33°	34°	35°	36°	39°	41°	
Diaspididae	*Aonidiella aurantii*	—	—	—	—	3	—	—	—	—	—	—	9
	*Aonidiella ensifera*	—	—	—	2	2	2	—	—	—	—	—	14
	*Aonidomytilus sp*.	—	—	—	—	—	—	—	—	—	1	1	4
	*Aspidiotus nerii* I	—	1	—	2	5	1	3	2	2	—	—	37
	*Aspidiotus nerii* II	—	—	—	3	4	3	—	—	—	—	—	15
	*Aspidiotus nerii* III	—	1	2	6	8	1	—	1	1	—	1	49
	*Chrysomphalus dictyospermi*	—	—	—	—	—	—	1	—	—	—	—	6
	*Diaspidiotus ancylus*	—	—	—	1	—	—	—	1	—	—	—	2
	*Diaspidiotus perniciosus*	—	—	1	3	2	2	1	—	—	—	—	32
	*Diaspis chilensis*	—	—	—	—	2	—	1	—	—	—	—	7
	*Diaspis echinocacti*	—	—	—	—	1	—	—	—	—	—	—	1
	*Epidiaspis leperii*	—	—	—	—	—	1	—	1	1	—	—	5
	*Furchadaspis zamiae*	—	—	—	—	1	—	—	—	—	—	—	2
	*Hemiberlesia lataniae*	—	—	—	4	10	1	1	—	—	—	—	51
	*Hemiberlesia palmae*	1	—	—	—	—	—	—	—	—	—	—	1
	*Hemiberlesia rapax* I	—	—	1	1	2	1	—	1	2	—	—	14
	*Hemiberlesia rapax* II	1	1	—	1	2	—	1	1	—	—	—	9
	*Hemiberlesia rapax* III	—	—	—	2	2	—	—	1	1	—	—	7
	*Lepidosaphes beckii*	—	—	1	3	5	—	1	—	—	—	—	19
	*Lepidosaphes ulmi*	—	—	—	2	1	3	—	—	—	—	—	9
	*Melanaspis sitreana*	—	—	—	—	2	—	—	—	—	—	—	4
	*Pseudoparlatoria chilina*	—	—	—	—	—	—	—	—	—	1	—	2
	*Pseudoparlatoria sp*.	—	—	—	—	—	—	—	—	—	1	—	3
Coccidae	*Ceroplastes sinensis*	—	—	—	—	3	—	—	1	—	—	—	8
	*Ceroplastes sp*. I	—	—	—	—	1	—	—	—	1	—	—	3
	*Ceroplastes sp*. II	—	1	—	—	—	—	—	—	—	—	—	1
	*Unidentified Coccidae*	—	—	—	1	—	—	—	—	—	—	—	2
	*Coccus hesperidum*	—	1	—	4	6	2	—	—	1	1	—	24
	*Parasaissetia nigra*	—	—	—	1	—	—	—	—	—	—	—	2
	*Parthenolecanium corni*	—	—	—	—	2	—	—	1	—	—	—	6
	*Protopulvinaria pyriformis*	—	—	—	2	9	1	1	—	—	—	—	32
	*Pulvinariella mesembryanthemi*	—	—	—	—	—	—	—	—	—	1	1	6
	*Saissetia coffeae*	1	1	1	2	—	—	—	—	—	—	—	11
	*Saissetia oleae*	—	1	1	2	19	9	9	6	3	—	—	158

The values in the table indicate the number of sites sampled per region. The minimum distance between sites was at least 1 km. Latitudes correspond to the median coordinate of each administrative region.

### Diaspididae

Seventeen Diaspididae species were identified morphologically: *Aonidiella aurantii* (Maskell), *Aonidiella ensifera* McKenzie, *Aspidiotus nerii* Bouché, *Chrysomphalus dictyospermi* (Morgan), *Diaspidiotus ancylus* Putnam, *Diaspidiotus echinocacti* (Bouché), *Diaspidiotus perniciosus* (Comstock), *Diaspis chilensis* Cockerell, *Epidiaspis leperii* Signoret, *Furchadaspis zamiae* (Morgan), *Hemiberlesia lataniae* (Signoret), *Hemiberlesia rapax* (Comstock), *Hemiberlesia palmae* (Cockerell), *Lepidosaphes beckii* (Newman), *Lepidosaphes ulmi* (Linnaeus), *Melanaspis sitreana* (Hempel), *Pseudoparlatoria chilina* (Lindinger). Two other members of the Diaspididae were not identified to species level. One belonged to genus *Pseudoparlatoria* and the other belonged to genus *Aonidomytilus*.

From 343 individuals of Diaspididae processed, 268 (78%) unambiguous sequences were obtained for COI and 275 (80%) unambiguous sequences were obtained for 28S. In total, at least one gene was successfully sequenced for 302 individuals and both genes were successfully sequenced for 241 individuals.

For COI, 40 different haplotypes were found. The length of the sequence alignment was 649 bp for 36 haplotypes. Four haplotypes of three species differed: both ends of the sequences of *Furchadaspis zamiae* were unreadable (resulting in a sequence length of 445 bp – HCOI11 and HCOI12). The sequences of *Hemiberlesia rapax* III presented an insertion of 24 repetitions of ‘TAA’ and had length of 656 bp. The sequences of *Aspidiotus nerii* III presented two insertions of 227 bp and 4 bp and had length of 880 bp. Both sequences were suspected to be COI-like pseudogene and they were not taken into account in the following analyses. All COI sequences had a bias toward low GC content (A = 39.8%, T = 41.6%, C = 11.9% and G = 6.7%), with a mean GC content of about 18.6% (range: 16.9–20.0%). A neighbor-joining tree was plotted for COI (Fig. 1, also including the Coccidae). Two monophyletic lineages of *Aspidiotus nerii* (I and II) and a complex of two cryptic species of *Hemiberlesia rapax* (I and II) were observed. For 28S, we identified 34 different haplotypes. The length of high-quality sequence ranged from 706 bp to 720 bp for species of the tribe Diaspidini and from 721 to 736 bp for species of the tribe Aspidiotini. A neighbor-joining tree was plotted for 28S (Fig. 2, also including the Coccidae). The initial ABGD partition delineated 19 groups within the Diaspididae due to the subdivision of *H*. *rapax* into two groups and the unsuccessful PCR for *Pseudoparlatoria* sp. The recursive partition delineated 23 groups due to the subdivision of *H*. *lataniae* and *D*. *chilensis* into two groups, and *A*. *nerii* into three groups.

We determined the distribution of the intraspecific (black) and interspecific (gray) pairwise distances according to the K2P nucleotide substitution model (Fig. 3B). For these analyses, we used 19 species for 28S and 20 species for COI. Both *Hemiberlesia rapax* and *Aspidiotus nerii* was subdivided into two groups. A barcode gap was observed for intra- and interspecific distances, for both genes. The intraspecific distance ranged from 0% to 1.7% for COI and from 0% to 0.4% for 28S. The interspecific distance ranged from 6.3% to 27.9% for COI and from 1.5% to 17.4% for 28S. The interspecific distance for COI interspecific distance ranged from 7.7% to 8.2% within the two linages of *A*. *nerii*, and from 6.3% to 6.5% within the cryptic species of *H*. *rapax*. The 28S interspecific distance ranged from 0 to 0.01% within the two linages of *A*. *nerii*, and was 0.3% within the cryptic species of *H*. *rapax*.

For 18 out of 40 COI haplotypes, the species identification corresponded to the scientific names of the best GenBank hit with a percentage of similarity over 90.8% (Supplementary Data 2). The lowest values corresponded to the haplotypes of *Aspidiotus nerii* I (~93%) and *Hemiberlesia lataniae* (~91%). Without taking into account these haplotypes, the percentage of similarity was over 98.5%. For 20 out of 34 28S haplotypes, the species identification corresponded to the scientific names of the best GenBank hit with a percentage of similarity over 99.3%. Three GenBank hits mismatched with a percentage of similarity higher than this threshold: *Aonidomytilus* sp. matched with *Dactylaspis* sp. (100%); *Aonidiella aurantii* with *Aonidiella* sp. (100%) and *Hemiberlesia palmae* with *Abgrallapsis cyanophylli* (99.4%). The result of Blastn request for each haplotype is provided in Supplementary Data 2.

Each lineage of *A*. *nerii* had specific COI haplotypes, but shared at least the haplotype H28S33 (n = 83). Individuals of *A*. *nerii* I (n = 33) displayed the haplotype HCOI28 or HCOI29. Individuals of *A*. *nerii* II (n = 49) displayed five haplotypes: HCOI30 to HCOI34. Individuals of *A*. *nerii* III (n = 15) had the COI-like pseudogene. Contrary to *A*. *nerii*, each cryptic species of *H*. *rapax* complex displayed specific haplotypes of COI and 28S. Individuals of *H*. *rapax* I (n = 13) displayed the haplotype HCOI21 and the haplotypes H28S20 or H28S21. No individuals of *H*. *rapax* II (n = 9) were sequenced for both genes and they displayed the haplotypes HCOI37 to HCOI39, or the haplotype H28S15. Individuals of *H*. *rapax* III (n = 7) with the COI-like pseudogene displayed the haplotypes H28S16 to H28S19.

The Diaspididae with the widest distribution were *Aspidiotus nerii* sampled from 19°S to 41°S and *Hemiberlesia rapax* sampled from 18°S to 36°S (Table 1). Lineage I and II of *A*. *nerii* had comparable distribution from 19° to 33°S (up to 510 m above sea level) and from 20° to 41°S (up to 1280 m above sea level) respectively, while the lineage III (COI-like pseudogene) of *A*. *nerii* ranged from 29° to 33°S (from 120 to 790 m above sea level). Each cryptic species of *H*. *rapax* displayed different geographical patterns: *H*. *rapax* I was sampled from 28°S to 36°S (up to 540 m above sea level); *H*. *rapax* II from 18° to 35°S (up to 830 m above sea level); *H*. *rapax* III (COI-like pseudogene) from 30° to 36°S (from 130 to 1180 m above sea level). The other species were found on the central zone of Chile (29°S to 34°S, Regions IV to VI) except the genus *Pseudoparlatoria* and *Aonidomytilus* only found in the southern regions (from 39° to 41°S) and *H*. *palmae* only found in the North of Chile (18°S).

The Diaspididae found on the greatest number of host plants were *Aspidiotus nerii* and *Hemiberlesia rapax* (considering all three groups for both species), collected on 18 host plants (from nine orders) and 13 host plants (from seven orders), respectively (Supplementary Data 1 and 3). Within the three lineages of *A*. *nerii*, *A*. *nerii* I was found on seven host plants among which *Aristotelia chilensis* (Elaeocarpaceae) and *Persea americana* (Lauraceae) (9 out of 10 sites) were specific to this first lineage. *A*. *nerii* II was found on 13 host-plants among which six were specific (*Citrus spp*. (Rutaceae), *Annona cherimola* (Annonaceae), *Macadamia* sp. (Proteaceae), *Nerium oleander* (Apocynaceae), *Quillaja saponaria* (Quillajaceae), *Peumus boldo* (Monimiaceae)) to this second lineage. *A*. *nerii* III was found on six ornamental and South American trees among which *Euonymus sp*. (Celastraceae) and *Schinus molle* were specific to this third lineage. Within the cryptic species of *H*. *rapax*, *H*. *rapax* I was found on 6 host-plants among which *Citrus sinensis* and *Nerium oleander* were specific to this first cryptic species. *H*. *rapax* II found on 6 host-plants among which only *Acacia retinodes* (Fabaceae) was specific to this second cryptic species. *H*. *rapax* III was found on five host-plants each shared with at least one of the other cryptic species. The complete list of host plants for each species is provided in Supplementary Data 1 and 3 (by host plant family and order).

## Discussion

This survey reported COI and 28S barcode sequences for 11 putative species of Coccidae and 19 of Diaspididae from Chile, among which eight Coccidae and 17 Diaspididae species could be assigned a name based on morphological examination. Except for *Parasaissetia nigra* which is a new mention for Chile, all these species were already reported in the country^21, 26–28^. Three species of Coccidae could not be morphologically identified because only immature stages were collected and the GenBank matches were not associated with high similarity percentages. Two species of Diaspididae were not morphologically identified at species levels and belonged to the genus *Pseudoparlatoria* and *Aonidomytilus*, and were found on native plant hosts. In Chile, only *Aonidomytilus espinosai* (Porter) is mentioned for this genus^1^. The K2P distance between the 28S sequences of *A*. *espinosai* (GenBank accession: DQ145290 and DQ145291) and the haplotype H28S07 was 7.1%. This value is superior to the maximum intraspecific distance observed (0.4%) but it is included within the range of the interspecific distance (from 1.5% to 17.4%). This species of *Aonidomytilus* is probably a new species for Chile. No reference sequence was available for *Pseudoparlatoria*. Four others endemic species of Diaspididae were collected: *Aonidiella ensifera*, *Diaspis chilensis*, *Melanaspis sitreana*, *Pseudoparlatoria chilina* ^26^. They were collected mainly on endemic plants, such as *Quillaja saponaria* or *Nothofagus* spp. (Nothofagaceae), and on *Hedera helix* (Araliaceae) and *Olea europaea* (Oleaceae) for *A*. *ensifera*. For Coccidae, sequences of the recently recorded *Pulvinariella mesembryanthemi* ^21^ were provided.

Most COI and 28S sequences are new barcodes for the morphologically identified species. More than 50% of haplotypes (39 for COI and 28 for 28S) match with sequences from the GenBank database with low percentage of similarity, probably due to the scarcity of sequence data for these groups, especially in South America. The previous molecular systematic studies included few individuals from Argentina, Colombia and Chile (n = 1) for Diaspididae^18, 19, 29, 30^ and from Colombia for Coccidae^31^. Futhermore, the COI region we sequenced (the Folmer region of COI, i.e. the “Barcode region”) does not overlap with the COI region sequenced in a set of previous studies, notably those using a fragment overlaping the 5′ end of COI and the 3′ end of COII. However, the low amount of GenBank matches at 28S can hardly be explained by the use of different 28S regions since the one sequenced in this study and in previous ones overlap over more than 650 bp. In addition, we noticed an inconsistency between the sequences deposited by Wei *et al*. (data deposited on GenBank without reference to a published article) as *Unaspis yanonensis* (Diaspididae) and the haplotypes HCOI16, HCOI18, HCOI19 and HCOI20 identified in our study as *Coccus hesperidum* (Coccidae). It is difficult to deal with inconsistencies between databases when all studies do not carry out morphological vouchering of specimens from which DNA has been extracted. To enable future revisions of the samples processed in this study, we deposited all our slide-mounted vouchers in the collections of ANSES (Montferrier sur Lez, France) and/or MNHN (Santiago, Chile).

These new sequences will be useful to future identification of these species. However, we noticed that the COI sequences gave more relevant information for the delineation of the *Ceroplastes* species as observed by Deng *et al*.^17^ on six *Ceroplastes* species from China. For COI, we obtained for each species one distinct haplotype while for 28S only two species were successfully sequenced. Moreover, the interspecific distance within COI haplotypes of the three species ranged from 12.5% to 17.5% which is superior to the minimum interspecific distance observed (12.5% - Fig. 3A) and congruent with the average intraspecific distance (12.2%) found by Deng *et al*.^17^. Overall, the combination of these COI and 28S markers has repeatedly proved useful for routine identification and DNA barcoding of Coccidae, Diaspididae and Pseudococcidae^13, 15, 32^, with 28S providing high rates of successful PCR and signal to easily distinguish most species and COI providing more polymorphism to distinguish closely related species and feed large DNA-barcodes databases relying on the COI Folmer region. However, for large-scale systematics studies, one may choose to switch to multiplex PCR using a set of mitochondrial and nuclear markers followed by next generation sequencing^33^.

Another result was the remarkably low G-C content observed in the COI gene for both Diaspididae (18.8%) and Coccidae (21.9%). This low G-C content of COI gene is common for the scale insects: 14.9% in Pseudococcidae^12^, 18.2% in Diaspididae^12^ and 20.4% in Ceroplastes^17^. This average content is lower than the value of 27.7–39.5% in other orders insects^9^.

For the Diaspididae, the oleander scale *A*. *nerii* and the greedy scale *H*. *rapax* displayed high diversity of haplotypes and contrasted geographical and host plant distribution patterns. Three lineages of *A*. *nerii* were observed at COI: the lineage I was found on 90% of the avocado trees whereas *Citrus* trees only hosted the lineage II; the lineage III (COI-like pseudogene) showed the smallest distribution within the central regions (temperate climate) of Chile. *A*. *nerii* is a cosmopolitan and polyphagous pest that comprises multiple cryptic parthenogenetic and sexual taxa^34, 35^. The Chilean diversity of *A*. *nerii* could be the result of local adaptation or of multiple introductions of the different lineages from various source populations. As parthenogenetic lineages are known to occur in the *A*. *nerii* complex^34, 35^, locating parthenogenetic lineages in Chile could be an advantage for national biocontrol manufacturers as rearing parthenogenetic scales generally enable higher population growth rates and subsequently more cost-efficient production of parasitoids. Further investigations should be dedicated to look for this trait within the Chilean lineages due to low-quality comparison with GenBank sequences. Direct observation or testing the presence of the endosymbiont *Cardinium* ^35^ could be two ways to detect parthenogenesis within *A*. *nerii* lineages. The cryptic species complex of *H*. *rapax* also displayed different lineages with distinct host distributions. *Citrus* trees only hosted *H*. *rapax* I while *H*. *rapax* III shared its host plants with at least another cryptic species. In contrast to *A*. *nerii*, the diversity of *H*. *rapax* could be explained by the fact that South America is the native region of the genus *Hemiberlesia* ^36^.

For the Coccidae, the brown scale, *Coccus hesperidum*, presented high diversity for COI with six haplotypes and an COI intraspecific distance up to 2.0% which was the maximum value obtained for this distance (Fig. 3A). Moreover, the initial ABGD partition suggested two groups: the group A (n = 16) with the haplotype HCOI48 to HCOI50 and the group B (n = 5) with the haplotypes HCOI45 to HCOI47 (Fig. 1). The group A had the greater distribution (20°S to 39°S) and number of host-plants (9 species) than the group B sampled from 29°S to 33°S on three host-plants. Furthermore, the unique haplotype of 28S matched at 99.3% with a sequence from *Coccus formicarii* (GenBank accession: JX866687). The study of Lin *et al*.^31^ suggested the genus *Coccus* might not be monophyletic. New studies of this group including South American samples may provide insights into the species delineation in this genus. The black scale, *Saissetia oleae*, also presented high diversity with six haplotypes for COI and nine haplotypes of 28S. The 28S intraspecific distance reached 0.6% which was the maximum value for this distance (Fig. 3A). The black scale is a polyphagous species, widely distributed around the world and one of the most notorious Coccidae pests in Chile^37, 38^. We collected this species in most surveyed regions (from 19°S to 36°S) and on more than 26 host plants. The haplotype H28S36 and H28S37 (Fig. 2) were not found on the northern regions of Chile (up to 32°S). However, few individuals sequenced came from these northern regions and we cannot conclude the existence of geographical pattern. Moreover, there is no haplotype pattern of host plant structuration for *Saissetia oleae*.

Occurrence of cryptic species or association of some lineages with certain host plants could have repercussion on their pest status and their biological control using parasitoids. Indeed, some hymenopteran parasitoids are known to be highly host specific^39, 40^ and consequently pest management through biocontrol may have to be adjusted for each lineage separately. Even though this study did not have the objective of determining pest species, we could establish an overview of the scale insects collected by crop. The complete list of hosts for each species is provided in Supplementary Data 1. Further studies, focused on each crop, are necessary to determine the scale insects species and their diversity in order to improve the biological control of these pests identifying adequate parasitoids and/or predators.

This study brings new barcode sequences from cosmopolitan and endemic species of Coccidae and Diaspididae from Chile. These new sequences of COI and 28S of South America are now available for further worldwide phylogenetic studies. One species, *Parasaissetia nigra* is a new mention for Chile. The study unraveled high diversity in the genera *Hemiberlesia* and also likely cryptic lineages within *Aspidiotus nerii* and *Coccus hesperidum*.

## Methods

### Sample collection

**Figure 4:**
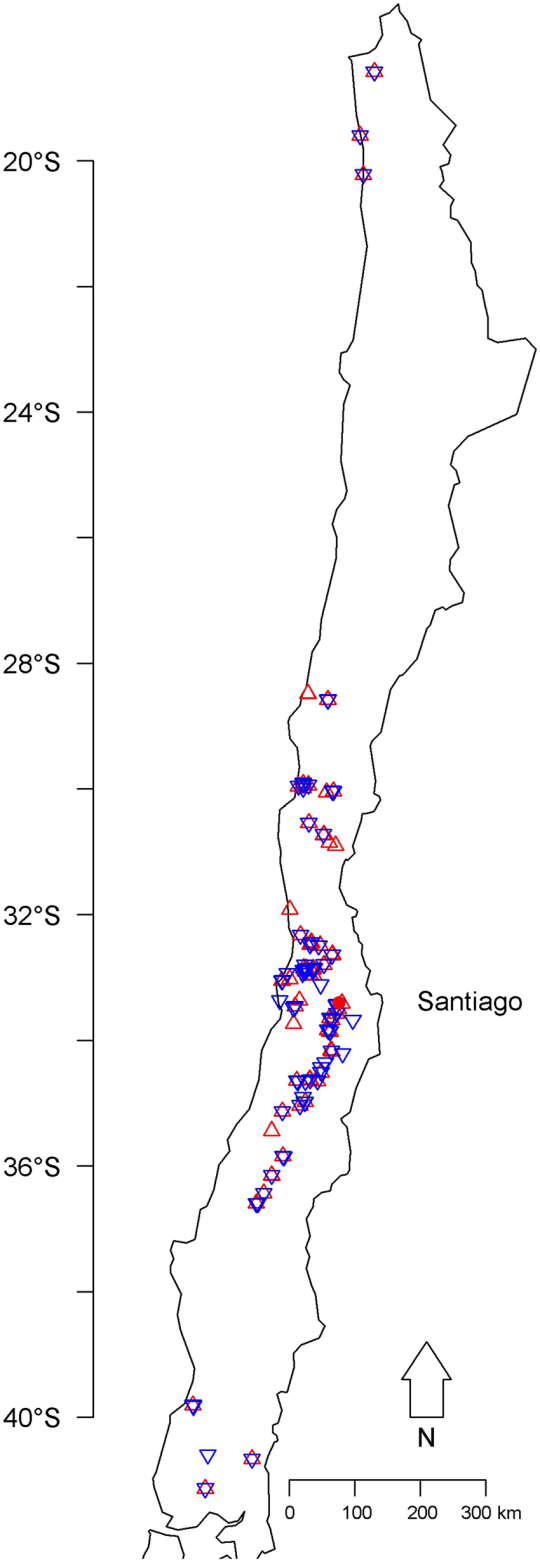
Sites sampled for Diaspididae (red upward-pointing triangle, *n* = 116) and for Coccidae (blue downward- pointing triangle, *n* = 112). Figure created using the software R^44^: library ‘maps’^56^.

In total, 116 populations of Diaspididae and 112 populations of Coccidae were sampled along a gradient running from Arica in northern Chile (18°S) to Frutillar in the south (41°S), between January 2015 and February 2016 (Fig. 4). The minimum distance between two sampled sites was 1 km. We collected up to 10 adult females from the Diaspididae or immature instars and young females from the Coccidae from crops, indigenous or endemic trees and shrubs and ornamental plants. The collected insects were preserved in 95% ethanol and stored at −20 °C for molecular analysis and morphological identification. Details about the samples collected including sampling locations, host plants, and dates, are available in Supplementary Data 1.

### DNA extraction and amplification

When possible, we analyzed two individuals from each sample collected. DNA was extracted from 343 specimens of Diaspididae and 293 of Coccidae, with the DNeasy Tissue Kit (QIAGEN, Hilden, Germany). The extraction was performed without crushing the body of the insect, and it was therefore possible to recover the specimen for its subsequent morphological identification. The procedure used followed the manufacturer’s guidelines, but with two minor changes to improve DNA extraction: cell lysis was carried out over a period of six to eight hours, and we used two elution steps (2 × 50 μl of AE buffer)^32^.

We amplified two different loci, chosen for analysis on the basis of their suitability for DNA barcoding, population genetics and phylogenetic studies: the HCO-LCO region of the mitochondrial locus encoding cytochrome oxidase subunit I (COI) and the D2 region of the nuclear 28S gene^32^. For COI, we used the primers PCO-F1 (5′ CCTTCAACTAATCATAAAAATATYAG 3′) and Lep-R1 (5′ TAAACTTCTGGATGTCCAAAAAATCA 3′); and for 28S, we used the primers C-28SLong-F (5′ GAGAGTTMAASAGTACGTGAAAC 3′) and C-28SLong-R (5′ TCGGARGGAACCAGCTACTA 3′). PCR was performed with a 23 μl reaction mixture and 2 μl of diluted DNA (1–20 ng). The reaction mixture contained 12.5 μl of 1X QIAGEN Multiplex PCR buffer and each of the primers required at a concentration of 0.2 μM. PCR was carried out as follows: initial denaturation at 95 °C for 15 minutes, followed by 35 cycles of denaturation at 94 °C for 30 s, annealing for 90 s at a temperature of 48 °C (COI) or 58 °C (28S), depending on the primers, elongation at 72 °C for 60 s, and then a final extension phase at 72 °C for 10 minutes. The final products were separated by electrophoresis with the QIAxcel Advanced System (QIAGEN, Hilden, Germany) for quality control. The, PCR products were then sent to Genewiz (UK, Essex), for bidirectional sequencing by capillary electrophoresis on an ABI 3130XL automatic sequencer (Applied Biosystems, Foster City, CA, USA). The consensus sequences were provided by Genewiz. Chromatograms were visualized with SeqTrace 0.9.0^41^ to check nucleotide variations. The sequences were deposited in GenBank (COI accession numbers: KY084933 to KY085394 and 28S accession numbers: KY085395 to KY085889 – Supplementary Data 1).

### Phylogenetic analysis

We considered sequences differing by one or more nucleotides to correspond to different haplotypes. Haplotype alignment was performed with MEGA version 7^42^ with the CLUSTALW method^43^. Blastn queries against NCBI GenBank, neighbor-joining tree generation and bootstrapping were performed on the haplotypes of each gene with the R^44^: libraries “ape”^45^, “ade4”^46^, and “phangorn”^47^. The intraspecific and interspecific pairwise distances were calculated with the R package ‘adhoc’^48^. The Kimura two-parameter nucleotide substitution model (K2P)^49^ was used for all analyses. Species delineation was performed on the concatened 28S and COI sequences with the online version of Automatic Barcode Gap Discovery (ABGD)^50^, with a prior maximal distance P = 0.001 and a Jukes-Cantor MinSlope distance of 1.0.

### Morphological identification

In the laboratory, we mounted one to ten specimens per COI and 28S haplotype on slides, as follows: specimens were macerated in 10% KOH, washed in distilled water, stained in a mixture of fuschin acid, distilled water, glycerol and lactic acid, dehydrated in acetic acid and lavender oil and mounted in Canada balsam^32^. All the slide-mounted specimens were deposited in the ANSES collection at Montferrier-sur-Lez and most were also deposited in the entomological collection of the Chilean National Museum of Natural History (MNHN) of Santiago, Chile (Supplementary Data 1). Several taxonomic and ecological articles were used for the identification of species and establishment of their distribution^37, 51–55^.

## Electronic supplementary material

Supplementary Data 1, 2 and 3
