# Supplementary material for: Genetic diversity of armored scales (Hemiptera: Diaspididae) and soft scales (Hemiptera: Coccidae) in Chile
Source: Sci Rep. 2017 May 17;7:2014. doi: 10.1038/s41598-017-01997-6 (PMC5435716; doi:10.1038/s41598-017-01997-6)
Supplement: Supplementary file 1 — Supplementary Data 1, 2 and 3 [file 41598_2017_1997_MOESM1_ESM.pdf]

SUPPLEMENTARY DATA

Genetic diversity of armored scales (Hemiptera: Diaspididae) and soft scales (Hemiptera: Coccidae) in Chile

Amouroux, P.[1*], Crochard D.[2], Germain J-F[3], Correa M.[2], Ampuero J.[4], Groussier G.[2], Kreiter P.[2], Malausa, T.[2] & Zaviezo, T.[1]

Supplementary Data 1

List, in alphabetical order, of the 555 individuals sequenced, their sample date and location (administrative region, municipality, longitude and latitude, elevation), their host plant (scientific name), the haplotype code for COI and 28S, the voucher location, the molecular code for the voucher in the INRA collection, the voucher code (corresponding to individuals morphologically identified) for the voucher in the MNHN and ANSES collections.

Supplementary Data 2

Result for the 54 haplotypes of 28S and 70 haplotypes of COI for best hits for Blastn queries of the NCBI GenBank database, by family and in alphabetical order (Accessed September 2016). The Blastn options were: *sscinames*: subject scientific name; *length*: alignment length; *sseqid*: subject sequence ID; *evalue*: expected value; *bitscore*: Bit-score; *qcovs*: query coverage per subject (for all HSPs); *pident*: percentage of identical matches.

Supplementary Data 3

List, by family and alphabetical order, of the 21 species of Diaspididae and 13 species of Coccidae surveyed, with their numbers of host plant species, grouped by order and family. Host plant order was sorted (left to right) according to the Angiosperm Phylogeny Website (http://www.mobot.org/MOBOT/research/APweb/welcome.html). (Accessed September 2016)

Species name	Family	Sample Date	Host plant	Type	Region	Municipality	Longitude	Latitude	Altitude (m.)	Area	Haplotype COI	Haplotype 28S	Molecular Code	Voucher Location	Voucher Code	GenBank access. COI	GenBank access. 28S
Aonidiella aurantii	D	Apr-15	*Citrus sinensis*	I	V	Quillota	-71,192228	-32,871839	174	C	HCOI26	H28S24	19428	ANSES	1600214	KY084992	KY085494
Aonidiella aurantii	D	Apr-15	*Citrus sinensis*	I	V	Quillota	-71,192228	-32,871839	174	C	HCOI26	-	19429	ANSES	1600214	KY084993	-
Aonidiella aurantii	D	Apr-15	*Citrus sinensis*	I	V	Quillota	-71,195847	-32,873494	158	C	HCOI26	H28S24	19544	INRA	-	KY085084	KY085497
Aonidiella aurantii	D	Apr-15	*Citrus sinensis*	I	V	Quillota	-71,194831	-32,874394	160	C	HCOI26	H28S24	19643	INRA	-	KY085175	KY085492
Aonidiella aurantii	D	Apr-15	*Citrus sinensis*	I	V	Quillota	-71,194831	-32,874394	160	C	HCOI25	H28S24	19642	INRA	-	KY085174	KY085493
Aonidiella aurantii	D	Feb-15	*Citrus limon*	I	V	Viña del mar	-71,490508	-33,019378	270	U	HCOI26	H28S24	19515	INRA	-	KY085062	KY085495
Aonidiella aurantii	D	Feb-15	*Citrus limon*	I	V	Viña del mar	-71,490508	-33,019378	270	U	HCOI26	H28S24	19514	INRA	-	KY085061	KY085498
Aonidiella aurantii	D	Feb-15	*Citrus limon*	I	V	Valparaiso	-71,630556	-33,047500	114	U	HCOI26	H28S24	19507	INRA	-	KY085055	KY085496
Aonidiella aurantii	D	Feb-15	*Citrus limon*	I	V	Valparaiso	-71,630556	-33,047500	114	U	HCOI26	H28S24	19506	MNHN	1600310	KY085054	KY085499
Aonidiella ensifera	D	Feb-16	*Vaccinium corymbosum*	I	IV	La Serena	-71,242586	-29,919058	92	C	HCOI36	H28S13	24333	INRA	-	KY085355	KY085447
Aonidiella ensifera	D	Feb-16	*Vaccinium corymbosum*	I	IV	La Serena	-71,242586	-29,919058	92	C	HCOI36	H28S13	24334	INRA	-	KY085356	KY085451
Aonidiella ensifera	D	May-15	*Olea europaea*	I	IV	Coquimbo	-71,336386	-29,955581	6	U	HCOI36	H28S14	19391	INRA	-	KY084959	KY085454
Aonidiella ensifera	D	May-15	*Olea europaea*	I	IV	Coquimbo	-71,336386	-29,955581	6	U	HCOI36	H28S14	19423	INRA	-	KY084987	KY085459
Aonidiella ensifera	D	Sep-15	*Hedera helix*	I	V	Quillota	-71,192197	-32,875308	167	C	HCOI36	H28S13	24266	MNHN	1600311	KY085313	KY085453
Aonidiella ensifera	D	Sep-15	*Hedera helix*	I	V	Quillota	-71,192197	-32,875308	167	C	HCOI36	H28S13	24267	INRA	-	KY085314	KY085450
Aonidiella ensifera	D	Apr-15	*Hedera helix*	I	V	Quillota	-71,209356	-32,896217	140	U	HCOI36	H28S14	19448	ANSES	1600212	KY085010	KY085457
Aonidiella ensifera	D	Apr-15	*Hedera helix*	I	V	Quillota	-71,209356	-32,896217	140	U	HCOI36	H28S14	19449	ANSES	1600212	KY085011	KY085458
Aonidiella ensifera	D	Mar-15	Unknow	-	Met	Nueva Independencia	-70,762842	-33,643031	510	U	HCOI36	H28S14	19533	INRA	-	KY085074	KY085460
Aonidiella ensifera	D	Jan-16	*Hedera helix*	I	Met	Paine	-70,755939	-33,855500	378	U	HCOI36	H28S13	24323	INRA	-	KY085350	KY085448
Aonidiella ensifera	D	Jan-16	*Hedera helix*	I	Met	Paine	-70,755939	-33,855500	378	U	-	H28S13	24324	INRA	-	-	KY085452
Aonidiella ensifera	D	Oct-15	*Hedera helix*	I	Met	Paine	-70,758053	-33,855528	378	U	-	H28S13	24281	INRA	-	-	KY085449
Aonidiella ensifera	D	Oct-15	*Hedera helix*	I	Met	Paine	-70,758053	-33,855528	378	U	-	H28S14	27885	INRA	-	-	KY085456
Aonidiella ensifera	D	Oct-15	*Hedera helix*	I	Met	Paine	-70,758053	-33,855528	378	U	-	H28S14	27886	INRA	-	-	KY085455
Aonidomytilus sp.	D	Nov-15	Unknow	-	XIV	Valdivia	-73,251028	-39,806264	18	U	HCOI10	H28S07	24302	ANSES	1600297	KY085336	KY085429
Aonidomytilus sp.	D	Nov-15	Unknow	-	XIV	Valdivia	-73,251028	-39,806264	18	U	HCOI10	H28S07	24301	ANSES	160097	KY085335	KY085428
Aonidomytilus sp.	D	Nov-15	*Ilex sp.*	I	X	Frutillar	-73,026983	-41,135564	58	U	HCOI09	H28S07	24303	INRA	-	KY085337	KY085426
Aonidomytilus sp.	D	Nov-15	*Ilex sp.*	I	X	Frutillar	-73,026983	-41,135564	58	U	HCOI09	H28S07	24304	INRA	-	KY085338	KY085427
Aspidiotus nerii	D	Jun-15	*Olea europaea*	I	IV	Ovalle	-71,144531	-30,537914	286	C	-	H28S33	23798	INRA	-	-	KY085589
Aspidiotus nerii	D	Jun-15	*Olea europaea*	I	IV	Ovalle	-71,144531	-30,537914	286	C	-	H28S33	23799	INRA	-	-	KY085588
Aspidiotus nerii	D	Jun-15	*Olea europaea*	I	IV	Ovalle	-71,144531	-30,537914	286	C	-	H28S33	27931	INRA	-	-	KY085587
Aspidiotus nerii	D	May-15	*Ilex sp.*	I	VIII	San Carlos	-71,962550	-36,434606	185	U	-	H28S33	19397	INRA	-	-	KY085635
Aspidiotus nerii I	D	Jul-15	*Acacia retinodes*	I	I	Pisagua	-70,211583	-19,596589	12	U	HCOI28	H28S33	24168	INRA	-	KY085257	KY085663
Aspidiotus nerii I	D	Jun-15	*Persea americana*	I	IV	Ovalle	-71,144531	-30,537914	286	C	HCOI29	H28S33	19566	INRA	-	KY085103	KY085592
Aspidiotus nerii I	D	Jun-15	*Persea americana*	I	IV	Ovalle	-71,144531	-30,537914	286	C	HCOI29	H28S32	24129	INRA	-	KY085252	KY085578
Aspidiotus nerii I	D	Apr-15	*Persea americana*	I	V	Cabildo	-71,097144	-32,439528	173	C	HCOI29	H28S32	19508	INRA	-	KY085056	KY085577
Aspidiotus nerii I	D	Sep-15	*Persea americana*	I	V	Cabildo	-71,098258	-32,440803	171	C	HCOI29	H28S33	24280	INRA	-	KY085324	KY085645
Aspidiotus nerii I	D	Sep-15	*Persea americana*	I	V	Cabildo	-71,098258	-32,440803	171	C	HCOI29	H28S33	24279	INRA	-	KY085323	KY085644
Aspidiotus nerii I	D	Mar-15	*Persea americana*	I	V	Cabildo	-71,094369	-32,445361	159	C	HCOI29	H28S33	19516	MNHN	1600206	KY085063	KY085600
Aspidiotus nerii I	D	Mar-15	*Persea americana*	I	V	Cabildo	-71,094369	-32,445361	159	C	HCOI29	H28S33	19517	MNHN	1600206	KY085064	KY085591
Aspidiotus nerii I	D	Apr-15	*Persea americana*	I	V	Cabildo	-71,093250	-32,446242	159	C	HCOI29	H28S32	19525	INRA	-	KY085070	KY085580
Aspidiotus nerii I	D	Apr-15	*Persea americana*	I	V	La Cruz	-71,190689	-32,855581	176	C	HCOI29	H28S33	19466	INRA	-	KY085024	KY085613
Aspidiotus nerii I	D	Sep-15	*Persea americana*	I	V	Hijuelas	-71,063264	-32,859917	407	C	HCOI29	H28S33	24274	INRA	-	KY085320	KY085653
Aspidiotus nerii I	D	Sep-15	*Persea americana*	I	V	Hijuelas	-71,063264	-32,859917	407	C	HCOI29	H28S32	24275	INRA	-	KY085321	KY085584
Aspidiotus nerii I	D	Apr-15	*Persea americana*	I	V	Hijuelas	-71,060967	-32,863178	506	C	HCOI29	H28S32	19456	INRA	-	KY085018	KY085582
Aspidiotus nerii I	D	Apr-15	*Persea americana*	I	V	Hijuelas	-71,060967	-32,863178	506	C	HCOI29	H28S32	19457	INRA	-	KY085019	KY085581
Aspidiotus nerii I	D	Sep-15	*Persea americana*	I	V	Quillota	-71,186689	-32,864986	185	C	HCOI29	-	24181	INRA	-	KY085266	-
Aspidiotus nerii I	D	Sep-15	*Persea americana*	I	V	Quillota	-71,186689	-32,864986	185	C	HCOI29	H28S33	24180	INRA	-	KY085265	KY085651
Aspidiotus nerii I	D	May-15	*Cryptocarya alba*	N1	Met	Paine	-70,795786	-33,833233	376	C	HCOI29	H28S33	19539	INRA	-	KY085080	KY085605

Species name	Family	Sample Date	Host plant	Type	Region	Municipality	Longitude	Latitude	Altitude (m.)	Area	Haplotype COI	Haplotype 28S	Molecular Code	Voucher Location	Voucher Code	GenBank access. COI	GenBank access. 28S
Aspidiotus nerii I	D	Oct-15	*Cryptocarya alba*	N1	VI	Rancagua	-70,726539	-34,170325	514	U	HCOI29	H28S33	24249	INRA	-	KY085299	KY085640
Aspidiotus nerii I	D	Oct-15	*Cryptocarya alba*	N1	VI	Rancagua	-70,726539	-34,170325	514	U	HCOI32	H28S33	24248	INRA	-	KY085298	KY085647
Aspidiotus nerii I	D	Oct-15	*Hedera helix*	I	VI	Santa Cruz	-71,364508	-34,638983	173	U	HCOI32	H28S33	24256	INRA	-	KY085304	KY085642
Aspidiotus nerii I	D	Oct-15	*Hedera helix*	I	VI	Santa Cruz	-71,364508	-34,638983	173	U	HCOI32	H28S32	24255	INRA	-	KY085303	KY085585
Aspidiotus nerii I	D	Feb-15	*Hedera helix*	I	VI	Chimbarongo	-70,988672	-34,642783	376	U	HCOI32	H28S33	19503	INRA	-	KY085051	KY085606
Aspidiotus nerii I	D	Feb-15	*Hedera helix*	I	VI	Chimbarongo	-70,988672	-34,642783	376	U	HCOI32	H28S33	19502	INRA	-	KY085050	KY085598
Aspidiotus nerii I	D	Mar-15	*Olea europaea*	I	VII	Sagrada Familia	-71,624278	-35,132542	96	C	HCOI32	H28S33	19479	INRA	-	KY085034	KY085609
Aspidiotus nerii I	D	Mar-15	*Olea europaea*	I	VII	Sagrada Familia	-71,624278	-35,132542	96	C	HCOI32	H28S33	19480	INRA	-	KY085035	KY085634
Aspidiotus nerii I	D	Mar-15	*Olea europaea*	I	VII	Pencahue	-71,820031	-35,437822	72	U	HCOI32	H28S33	19399	INRA	-	KY084964	KY085630
Aspidiotus nerii I	D	Mar-15	*Olea europaea*	I	VII	Pencahue	-71,820031	-35,437822	72	U	HCOI32	H28S32	19398	INRA	-	KY084963	KY085579
Aspidiotus nerii I	D	Apr-15	*Olea europaea*	I	VII	Pencahue	-71,820031	-35,437822	72	U	HCOI32	H28S34	19474	INRA	-	KY085031	KY085629
Aspidiotus nerii I	D	Apr-15	*Olea europaea*	I	VII	Pencahue	-71,820031	-35,437822	72	U	HCOI32	H28S33	19473	INRA	-	KY085030	KY085597
Aspidiotus nerii I	D	May-15	*Ilex sp.*	I	VIII	San Carlos	-71,962550	-36,434606	185	U	HCOI32	H28S32	19396	INRA	-	KY084962	KY085583
Aspidiotus nerii I	D	May-15	*Aristotelia chilensis*	N2	VIII	San Carlos	-71,962550	-36,434606	185	U	HCOI32	H28S33	19433	INRA	-	KY084997	KY085614
Aspidiotus nerii I	D	May-15	*Hedera helix*	I	VIII	San Carlos	-71,962550	-36,434606	185	U	HCOI32	H28S33	19403	INRA	-	KY084968	KY085590
Aspidiotus nerii I	D	May-15	*Hedera helix*	I	VIII	San Carlos	-71,962550	-36,434606	185	U	HCOI32	H28S33	19402	INRA	-	KY084967	KY085595
Aspidiotus nerii III	D	May-15	*Olea europaea*	I	IV	La Serena	-71,147150	-29,939536	120	U	Pseudogene	H28S33	19393	MNHN	1600219	KY084935	KY085603
Aspidiotus nerii III	D	May-15	*Olea europaea*	I	IV	La Serena	-71,147150	-29,939536	120	U	Pseudogene	H28S33	19392	MNHN	1600219	KY084933	KY085586
Aspidiotus nerii III	D	Jun-15	*Olea europaea*	I	IV	Ovalle	-71,144531	-30,537914	286	C	Pseudogene	-	24763	INRA	-	KY084946	-
Aspidiotus nerii III	D	Jun-15	*Schinus molle*	N1	IV	Monte Patria	-70,881442	-30,730389	565	C	Pseudogene	H28S33	19548	INRA	-	KY084945	KY085619
Aspidiotus nerii III	D	Sep-15	*Olea europaea*	I	V	Cabildo	-71,118781	-32,473328	133	U	Pseudogene	H28S33	24242	INRA	-	KY084938	KY085649
Aspidiotus nerii III	D	Sep-15	*Olea europaea*	I	V	Putaendo	-70,719083	-32,640931	790	U	Pseudogene	H28S33	24232	INRA	-	KY084941	KY085639
Aspidiotus nerii III	D	Apr-15	*Cryptocarya alba*	N1	V	La Cruz	-71,199806	-32,854186	146	C	Pseudogene	H28S33	19461	INRA	-	KY084934	KY085601
Aspidiotus nerii III	D	Apr-15	*Cryptocarya alba*	N1	V	La Cruz	-71,199806	-32,854186	146	C	Pseudogene	H28S33	19460	INRA	-	KY084936	KY085604
Aspidiotus nerii III	D	Apr-15	*Olea europaea*	I	V	La Cruz	-71,187997	-32,855800	195	C	Pseudogene	H28S33	19463	INRA	-	KY084943	KY085610
Aspidiotus nerii III	D	Apr-15	*Olea europaea*	I	V	La Cruz	-71,187997	-32,855800	195	C	Pseudogene	H28S33	19462	INRA	-	KY084944	KY085632
Aspidiotus nerii III	D	Mar-15	*Hedera helix*	I	Met	Las Condes	-70,540678	-33,408225	729	U	Pseudogene	H28S33	19528	INRA	-	KY084939	KY085636
Aspidiotus nerii III	D	Mar-15	*Euonymus sp.*	I	Met	San Bernando	-70,722472	-33,669439	544	U	Pseudogene	H28S33	19484	INRA	-	KY084942	KY085596
Aspidiotus nerii III	D	Mar-15	*Euonymus sp.*	I	Met	San Bernando	-70,722472	-33,669439	544	U	Pseudogene	H28S33	19485	INRA	-	KY084940	KY085631
Aspidiotus nerii III	D	Mar-15	*Aesculus hippocastanum*	I	Met	San Bernando	-70,722472	-33,669439	544	U	Pseudogene	H28S33	19487	INRA	-	KY084947	KY085617
Aspidiotus nerii III	D	May-15	*Cryptocarya alba*	N1	Met	Paine	-70,795786	-33,833233	376	C	Pseudogene	H28S33	19540	INRA	-	KY084937	KY085622
Aspidiotus nerii II	D	Jul-15	*Nerium oleander*	I	I	Iquique	-70,151092	-20,222303	12	U	HCOI34	-	24764	INRA	-	KY085389	-
Aspidiotus nerii II	D	Feb-16	*Olea europaea*	I	III	Huasco	-71,157900	-28,481622	37	C	HCOI33	H28S33	24361	INRA	-	KY085375	KY085665
Aspidiotus nerii II	D	Feb-16	*Olea europaea*	I	III	Huasco	-71,157900	-28,481622	37	C	HCOI33	H28S33	24362	INRA	-	KY085376	KY085669
Aspidiotus nerii II	D	Feb-16	*Olea europaea*	I	III	Huasco	-71,157900	-28,481622	37	C	HCOI33	H28S33	24363	INRA	-	KY085377	KY085667
Aspidiotus nerii II	D	Feb-16	*Nerium oleander*	I	III	Huasco	-71,156878	-28,483086	43	C	HCOI34	H28S33	24343	INRA	-	KY085364	KY085643
Aspidiotus nerii II	D	Feb-16	*Nerium oleander*	I	III	Huasco	-71,156878	-28,483086	43	C	HCOI34	H28S33	24344	INRA	-	KY085365	KY085664
Aspidiotus nerii II	D	Feb-16	*Olea europaea*	I	III	Vallenar	-70,795058	-28,578758	452	C	HCOI33	H28S33	24377	INRA	-	KY085386	KY085668
Aspidiotus nerii II	D	Feb-16	*Olea europaea*	I	III	Vallenar	-70,795058	-28,578758	452	C	HCOI34	H28S33	24376	INRA	-	KY085385	KY085650
Aspidiotus nerii II	D	Feb-16	*Macadamia sp.*	I	III	Vallenar	-70,797147	-28,581394	469	C	HCOI32	H28S33	24345	INRA	-	KY085366	KY085659
Aspidiotus nerii II	D	Feb-16	*Macadamia sp.*	I	III	Vallenar	-70,797147	-28,581394	469	C	HCOI34	H28S33	24346	INRA	-	KY085367	KY085657
Aspidiotus nerii II	D	Feb-16	*Annona cherimola*	N2	IV	La Serena	-71,242586	-29,919058	92	C	HCOI32	H28S33	24337	INRA	-	KY085359	KY085654
Aspidiotus nerii II	D	Feb-16	*Annona cherimola*	N2	IV	La Serena	-71,242586	-29,919058	92	C	HCOI30	H28S33	24338	INRA	-	KY085360	KY085646
Aspidiotus nerii II	D	Feb-16	*Citrus sinensis*	I	IV	Coquimbo	-71,250056	-29,984811	120	C	HCOI31	-	24369	INRA	-	KY085380	-
Aspidiotus nerii II	D	Feb-16	*Citrus sinensis*	I	IV	Coquimbo	-71,250056	-29,984811	120	C	HCOI31	H28S33	24370	INRA	-	KY085381	KY085666
Aspidiotus nerii II	D	May-15	*Olea europaea*	I	IV	Vicuña	-70,819556	-30,051969	589	N	HCOI32	H28S33	19418	INRA	-	KY084982	KY085621
Aspidiotus nerii II	D	May-15	*Olea europaea*	I	IV	Vicuña	-70,819556	-30,051969	589	N	HCOI32	H28S33	19419	INRA	-	KY084983	KY085612
Aspidiotus nerii II	D	Jun-15	*Citrus limon*	I	IV	Ovalle	-71,144531	-30,537914	286	C	HCOI32	-	19562	INRA	-	KY085099	-
Aspidiotus nerii II	D	Jun-15	*Citrus limon*	I	IV	Ovalle	-71,144531	-30,537914	286	C	HCOI32	H28S33	19561	INRA	-	KY085098	KY085623

Species name	Family	Sample Date	Host plant	Type	Region	Municipality	Longitude	Latitude	Altitude (m.)	Area	Haplotype COI	Haplotype 28S	Molecular Code	Voucher Location	Voucher Code	GenBank access. COI	GenBank access. 28S
Aspidiotus nerii II	D	Jun-15	*Persea americana*	I	IV	Ovalle	-71,144531	-30,537914	286	C	HCOI32	H28S33	19565	INRA	-	KY085102	KY085633
Aspidiotus nerii II	D	Jun-15	*Olea europaea*	I	IV	Monte Patria	-70,658025	-30,894922	1278	C	HCOI32	H28S33	19556	ANSES	1600226	KY085093	KY085625
Aspidiotus nerii II	D	Jun-15	*Peumus boldus*	N1	IV	Los vilos	-71,489075	-31,915369	70	U	HCOI32	H28S33	19542	INRA	-	KY085082	KY085607
Aspidiotus nerii II	D	Jun-15	*Peumus boldus*	N1	IV	Los vilos	-71,489075	-31,915369	70	U	HCOI32	H28S33	19541	INRA	-	KY085081	KY085616
Aspidiotus nerii II	D	Sep-15	*Olea europaea*	I	V	La Ligua	-71,295286	-32,321933	74	U	HCOI32	H28S33	24208	INRA	-	KY085280	KY085656
Aspidiotus nerii II	D	Sep-15	*Olea europaea*	I	V	La Ligua	-71,295286	-32,321933	74	U	HCOI32	H28S33	24207	INRA	-	KY085279	KY085658
Aspidiotus nerii II	D	Sep-15	*Olea europaea*	I	V	Cabildo	-71,118781	-32,473328	133	U	HCOI32	H28S33	24243	INRA	-	KY085295	KY085637
Aspidiotus nerii II	D	Sep-15	*Olea europaea*	I	V	Putaendo	-70,719083	-32,640931	790	U	HCOI32	H28S33	24233	INRA	-	KY085292	KY085655
Aspidiotus nerii II	D	Sep-15	*Nerium oleander*	I	V	Catemu	-70,869825	-32,791336	499	U	HCOI32	-	24226	INRA	-	KY085290	-
Aspidiotus nerii II	D	Sep-15	*Nerium oleander*	I	V	Catemu	-70,869825	-32,791336	499	U	HCOI32	H28S33	24227	INRA	-	KY085291	KY085662
Aspidiotus nerii II	D	Apr-15	*Prosopis sp.*	N2	V	La Cruz	-71,199806	-32,854186	146	C	HCOI32	H28S33	19454	INRA	-	KY085016	KY085594
Aspidiotus nerii II	D	Apr-15	*Prosopis sp.*	N2	V	La Cruz	-71,199806	-32,854186	146	C	HCOI32	H28S33	19455	INRA	-	KY085017	KY085615
Aspidiotus nerii II	D	Apr-15	*Olea europaea*	I	V	La Cruz	-71,190689	-32,855581	176	C	HCOI32	H28S33	19465	INRA	-	KY085023	KY085599
Aspidiotus nerii II	D	Apr-15	*Olea europaea*	I	V	La Cruz	-71,190689	-32,855581	176	C	HCOI32	H28S33	19464	INRA	-	KY085022	KY085593
Aspidiotus nerii II	D	Apr-15	*Prosopis sp.*	N2	V	Hijuelas	-71,064389	-32,856881	370	C	HCOI32	H28S33	19445	INRA	-	KY085007	KY085627
Aspidiotus nerii II	D	Apr-15	*Prosopis sp.*	N2	V	Hijuelas	-71,064389	-32,856881	370	C	HCOI32	H28S33	19444	INRA	-	KY085006	KY085624
Aspidiotus nerii II	D	Sep-15	*Citrus limon*	I	V	Hijuelas	-71,065958	-32,862044	425	C	HCOI32	H28S33	24223	INRA	-	KY085287	KY085660
Aspidiotus nerii II	D	Sep-15	*Citrus limon*	I	V	Hijuelas	-71,065958	-32,862044	425	C	HCOI32	H28S33	24224	INRA	-	KY085288	KY085661
Aspidiotus nerii II	D	Sep-15	*Citrus limon*	I	V	Hijuelas	-71,065958	-32,862044	425	C	HCOI32	-	24214	ANSES	1600291	KY085282	-
Aspidiotus nerii II	D	Sep-15	*Citrus limon*	I	V	Hijuelas	-71,065958	-32,862044	425	C	HCOI32	H28S33	24213	INRA	-	KY085281	KY085648
Aspidiotus nerii II	D	Sep-15	*Quillaja saponaria*	N1	V	Hijuelas	-71,075936	-32,866392	410	C	HCOI32	-	24189	INRA	-	KY085271	-
Aspidiotus nerii II	D	Sep-15	*Quillaja saponaria*	N1	V	Hijuelas	-71,075936	-32,866392	410	C	HCOI32	H28S33	24188	INRA	-	KY085270	KY085638
Aspidiotus nerii II	D	Mar-15	*Aesculus hippocastanum*	I	Met	San Bernando	-70,722472	-33,669439	544	U	HCOI32	H28S33	19486	INRA	-	KY085038	KY085626
Aspidiotus nerii II	D	Mar-15	*Nerium oleander*	I	Met	San Bernando	-70,722472	-33,669439	544	U	HCOI32	H28S33	19497	INRA	-	KY085046	KY085618
Aspidiotus nerii II	D	Mar-15	*Nerium oleander*	I	Met	San Bernando	-70,722472	-33,669439	544	U	HCOI32	H28S33	19496	INRA	-	KY085045	KY085602
Aspidiotus nerii II	D	May-15	*Nerium oleander*	I	VII	Parral	-71,821250	-36,149314	182	U	HCOI32	H28S33	19425	INRA	-	KY084989	KY085628
Aspidiotus nerii II	D	May-15	*Nerium oleander*	I	VII	Parral	-71,821250	-36,149314	182	U	HCOI32	H28S33	19424	INRA	-	KY084988	KY085608
Aspidiotus nerii II	D	May-15	*Acacia retinodes*	I	VIII	San Carlos	-71,962550	-36,434606	185	U	HCOI32	H28S33	19394	MNHN	1600223	KY084960	KY085620
Aspidiotus nerii II	D	May-15	*Acacia retinodes*	I	VIII	San Carlos	-71,962550	-36,434606	185	U	HCOI32	H28S33	19395	MNHN	1600223	KY084961	KY085611
Aspidiotus nerii II	D	Nov-15	*Ilex sp.*	I	X	Frutillar	-73,026983	-41,135564	58	U	HCOI34	H28S33	24309	INRA	-	KY085340	KY085652
Aspidiotus nerii II	D	Nov-15	*Ilex sp.*	I	X	Frutillar	-73,026983	-41,135564	58	U	HCOI34	H28S33	24310	INRA	-	KY085341	KY085641
Ceroplastes sinensis	C	Apr-15	*Citrus sinensis*	I	V	Quillota	-71,194831	-32,874394	160	C	HCOI53	H28S49	19662	INRA	-	KY085193	KY085822
Ceroplastes sinensis	C	Apr-15	*Pouteria lucuma*	N2	V	Quillota	-71,191833	-32,875178	170	C	HCOI53	H28S49	19653	INRA	-	KY085185	KY085826
Ceroplastes sinensis	C	Dec-15	*Citrus limon*	I	V	Quillota	-71,257778	-32,935056	230	C	HCOI53	-	24313	INRA	-	KY085342	-
Ceroplastes sinensis	C	Dec-15	*Citrus limon*	I	V	Quillota	-71,257778	-32,935056	230	C	HCOI53	-	24314	ANSES	1600309	KY085343	-
Ceroplastes sinensis	C	May-15	*Prunus persica*	I	V	Cartagena	-71,419708	-33,482900	218	C	HCOI53	H28S49	19655	ANSES	1600215	KY085187	KY085825
Ceroplastes sinensis	C	May-15	*Prunus persica*	I	V	Cartagena	-71,419708	-33,482900	218	C	HCOI53	H28S49	19654	ANSES	1600215	KY085186	KY085824
Ceroplastes sinensis	C	May-15	*Punica granatum*	I	VII	Linares	-71,600397	-35,848422	166	U	HCOI53	-	19582	INRA	-	KY085118	-
Ceroplastes sinensis	C	May-15	*Punica granatum*	I	VII	Linares	-71,600397	-35,848422	166	U	HCOI53	H28S49	19583	INRA	-	KY085119	KY085823
Ceroplastes sp. I	C	May-15	*Vaccinium corymbosum*	I	V	Casablanca	-71,396367	-33,456453	239	C	-	H28S48	19600	INRA	-	-	KY085821
Ceroplastes sp. I	C	May-15	*Berberis sp.*	I	VIII	Chillan	-72,071822	-36,593292	148	U	HCOI51	H28S47	19670	MNHN	1600221	KY085199	KY085820
Ceroplastes sp. I	C	May-15	*Berberis sp.*	I	VIII	Chillan	-72,071822	-36,593292	148	U	HCOI51	H28S47	19669	MNHN	1600221	KY085198	KY085819
Ceroplastes sp. II	C	Jul-15	*Schinus molle*	N1	I	Pisagua	-70,211583	-19,596589	12	U	HCOI52	-	24170	ANSES	1600289	KY085258	-
Chrysomphalus dictyospermi	D	Jan-16	*Hedera helix*	I	VI	Chimbarongo	-70,988950	-34,642528	374	U	HCOI35	H28S23	24320	INRA	-	KY085347	KY085486
Chrysomphalus dictyospermi	D	Jan-16	*Prunus laurocerasus*	I	VI	Chimbarongo	-70,988950	-34,642528	374	U	HCOI35	H28S23	24317	INRA	-	KY085344	KY085487
Chrysomphalus dictyospermi	D	Jan-16	*Prunus laurocerasus*	I	VI	Chimbarongo	-70,988950	-34,642528	374	U	HCOI35	H28S23	24318	INRA	-	KY085345	KY085490
Chrysomphalus dictyospermi	D	Jan-16	*Hedera helix*	I	VI	Chimbarongo	-70,988950	-34,642528	374	U	HCOI35	H28S23	24319	INRA	-	KY085346	KY085491
Chrysomphalus dictyospermi	D	Feb-15	*Hedera helix*	I	VI	Chimbarongo	-70,988672	-34,642783	376	U	HCOI35	H28S23	19489	MNHN	1600204	KY085040	KY085489
Chrysomphalus dictyospermi	D	Feb-15	*Hedera helix*	I	VI	Chimbarongo	-70,988672	-34,642783	376	U	HCOI35	H28S23	19488	MNHN	1600204	KY085039	KY085488

Species name	Family	Sample Date	Host plant	Type	Region	Municipality	Longitude	Latitude	Altitude (m.)	Area	Haplotype COI	Haplotype 28S	Molecular Code	Voucher Location	Voucher Code	GenBank access. COI	GenBank access. 28S
Coccidae unidentified	C	Feb-16	*Annona cherimola*	N2	IV	La Serena	-71,242586	-29,919058	92	C	HCOI41	-	24335	ANSES	1600293	KY085357	-
Coccidae unidentified	C	Feb-16	*Annona cherimola*	N2	IV	La Serena	-71,242586	-29,919058	92	C	HCOI41	-	24336	ANSES	1600293	KY085358	-
Coccus hesperidum	C	Jun-15	*Schinus molle*	N1	I	Iquique	-70,152108	-20,214975	16	U	HCOI48	H28S51	24163	INRA	-	KY085254	KY085841
Coccus hesperidum	C	Feb-16	*Citrus sinensis*	I	IV	La Serena	-71,242586	-29,919058	92	C	HCOI47	H28S51	24339	INRA	-	KY085361	KY085837
Coccus hesperidum	C	May-15	*Nerium oleander*	I	IV	Vicuña	-70,712817	-30,034611	616	U	HCOI45	H28S51	19615	INRA	-	KY085147	KY085829
Coccus hesperidum	C	May-15	*Nerium oleander*	I	IV	Vicuña	-70,712817	-30,034611	616	U	HCOI45	H28S51	19616	INRA	-	KY085148	KY085833
Coccus hesperidum	C	Feb-16	*Vitis vinifera*	I	IV	Vicuña	-70,696786	-30,035639	632	U	HCOI48	-	24358	INRA	-	KY085374	-
Coccus hesperidum	C	Feb-16	*Vitis vinifera*	I	IV	Vicuña	-70,696786	-30,035639	632	U	-	H28S51	24357	INRA	-	-	KY085842
Coccus hesperidum	C	Jun-15	*Citrus sp.*	I	IV	Monte Patria	-70,875686	-30,718414	505	C	HCOI50	H28S51	19724	ANSES	1600225	KY085251	KY085836
Coccus hesperidum	C	Jun-15	*Citrus sp.*	I	IV	Monte Patria	-70,875686	-30,718414	505	C	HCOI50	H28S51	19723	ANSES	1600225	KY085250	KY085835
Coccus hesperidum	C	Feb-16	*Acacia sp.*	I	V	La Calera	-71,214817	-32,798064	201	U	HCOI50	-	24767	INRA	-	KY085390	-
Coccus hesperidum	C	Feb-16	*Acacia sp.*	I	V	La Calera	-71,214817	-32,798064	201	U	-	H28S51	27927	INRA	-	-	KY085831
Coccus hesperidum	C	Feb-16	*Acacia sp.*	I	V	La Calera	-71,214817	-32,798064	201	U	-	H28S51	27928	INRA	-	-	KY085830
Coccus hesperidum	C	Aug-15	*Laurus nobilis*	I	V	Hijuelas	-71,042683	-32,856689	603	C	HCOI48	H28S51	24175	INRA	-	KY085261	KY085843
Coccus hesperidum	C	Aug-15	*Laurus nobilis*	I	V	Hijuelas	-71,042683	-32,856689	603	C	HCOI48	H28S51	24174	INRA	-	KY085260	KY085840
Coccus hesperidum	C	Sep-15	*Citrus limon*	I	V	Hijuelas	-71,065958	-32,862044	425	C	HCOI49	-	24225	INRA	-	KY085289	-
Coccus hesperidum	C	Sep-15	*Persea americana*	I	V	Quillota	-71,184086	-32,863000	201	C	HCOI49	H28S51	24263	INRA	-	KY085310	KY085845
Coccus hesperidum	C	Sep-15	*Quillaja saponaria*	N1	V	Hijuelas	-71,075936	-32,866392	410	C	HCOI46	H28S51	24196	INRA	-	KY085274	KY085839
Coccus hesperidum	C	Feb-15	*Citrus limon*	I	V	Valparaiso	-71,630556	-33,047500	114	U	HCOI46	-	19707	ANSES	1600230	KY085235	-
Coccus hesperidum	C	May-15	*Pyrus communis*	I	Met	Santiago	-70,633558	-33,569242	625	U	HCOI49	H28S51	19621	INRA	-	KY085153	KY085834
Coccus hesperidum	C	Oct-15	*Hedera helix*	I	Met	Paine	-70,758053	-33,855528	378	U	HCOI49	-	24251	INRA	-	KY085301	-
Coccus hesperidum	C	Oct-15	*Hedera helix*	I	Met	Paine	-70,758053	-33,855528	378	U	HCOI49	H28S51	24250	INRA	-	KY085300	KY085846
Coccus hesperidum	C	May-15	*Aristotelia chilensis*	N2	VIII	San Carlos	-71,962550	-36,434606	185	U	HCOI50	-	19595	INRA	-	KY085131	-
Coccus hesperidum	C	May-15	*Aristotelia chilensis*	N2	VIII	San Carlos	-71,962550	-36,434606	185	U	HCOI50	H28S51	19594	INRA	-	KY085130	KY085832
Coccus hesperidum	C	Nov-15	*Hedera helix*	I	XIV	Valdivia	-73,247578	-39,822711	7	U	HCOI49	H28S51	24292	INRA	-	KY085327	KY085838
Coccus hesperidum	C	Nov-15	*Hedera helix*	I	XIV	Valdivia	-73,247578	-39,822711	7	U	HCOI49	H28S51	24293	MNHN	1600308	KY085328	KY085844
Diaspidiotus ancylus	D	Jun-15	*Olea europaea*	I	IV	Monte Patria	-70,658025	-30,894922	1278	C	HCOI23	H28S27	19555	ANSES	1600226	KY085092	KY085530
Diaspidiotus ancylus	D	May-15	*Fraxinus sp.*	I	VII	Linares	-71,616908	-35,842264	156	U	HCOI22	H28S27	19538	INRA	-	KY085079	KY085529
Diaspidiotus perniciosus	D	Feb-16	*Prunus cerasus*	I	III	Vallenar	-70,797103	-28,581025	470	C	HCOI40	H28S25	24349	INRA	-	KY085368	KY085508
Diaspidiotus perniciosus	D	Feb-16	*Prunus cerasus*	I	III	Vallenar	-70,797103	-28,581025	470	C	HCOI40	H28S25	24350	INRA	-	KY085369	KY085507
Diaspidiotus perniciosus	D	Feb-16	*Prunus persica*	I	III	Vallenar	-70,797147	-28,581394	469	C	HCOI40	-	24351	INRA	-	KY085370	-
Diaspidiotus perniciosus	D	Feb-16	*Prunus persica*	I	III	Vallenar	-70,797147	-28,581394	469	C	HCOI40	H28S25	24352	INRA	-	KY085371	KY085506
Diaspidiotus perniciosus	D	Feb-16	*Prunus persica*	I	IV	Vicuña	-70,697019	-30,037658	632	U	HCOI40	H28S25	24354	INRA	-	KY085372	KY085509
Diaspidiotus perniciosus	D	Feb-16	*Prunus persica*	I	IV	Vicuña	-70,697019	-30,037658	632	U	-	H28S25	24353	INRA	-	-	KY085500
Diaspidiotus perniciosus	D	May-15	*Prunus persica*	I	IV	Vicuña	-70,697939	-30,038736	632	U	HCOI40	H28S26	19435	INRA	-	KY084999	KY085528
Diaspidiotus perniciosus	D	May-15	*Prunus persica*	I	IV	Vicuña	-70,697939	-30,038736	632	U	HCOI40	H28S25	19434	INRA	-	KY084998	KY085511
Diaspidiotus perniciosus	D	Jun-15	*Prunus persica*	I	IV	Monte Patria	-70,871358	-30,720767	563	C	HCOI40	H28S26	19564	INRA	-	KY085101	KY085526
Diaspidiotus perniciosus	D	Jun-15	*Prunus persica*	I	IV	Monte Patria	-70,871358	-30,720767	563	C	HCOI40	H28S26	19563	INRA	-	KY085100	KY085527
Diaspidiotus perniciosus	D	Jun-15	*Prunus persica*	I	IV	Monte Patria	-70,770686	-30,851267	715	C	HCOI40	H28S25	19560	INRA	-	KY085097	KY085510
Diaspidiotus perniciosus	D	Jun-15	*Prunus persica*	I	IV	Monte Patria	-70,770686	-30,851267	715	C	HCOI40	H28S25	19559	INRA	-	KY085096	KY085520
Diaspidiotus perniciosus	D	Apr-15	*Prunus persica*	I	V	Quillota	-71,192044	-32,875353	170	C	HCOI40	H28S25	19409	INRA	-	KY084974	KY085519
Diaspidiotus perniciosus	D	Apr-15	*Prunus persica*	I	V	Quillota	-71,192044	-32,875353	170	C	HCOI40	H28S25	19408	INRA	-	KY084973	KY085514
Diaspidiotus perniciosus	D	Dec-15	*Citrus limon*	I	V	Quillota	-71,257778	-32,935056	230	C	-	H28S25	25126	INRA	-	-	KY085503
Diaspidiotus perniciosus	D	May-15	*Cydonia oblonga*	I	Met	Santiago	-70,634269	-33,568869	623	U	HCOI40	H28S25	19401	INRA	-	KY084966	KY085524
Diaspidiotus perniciosus	D	May-15	*Cydonia oblonga*	I	Met	Santiago	-70,634269	-33,568869	623	U	HCOI40	H28S25	19400	INRA	-	KY084965	KY085517
Diaspidiotus perniciosus	D	May-15	*Malus domestica*	I	Met	La Pintana	-70,634269	-33,568869	623	U	HCOI40	H28S25	19411	INRA	-	KY084976	KY085513
Diaspidiotus perniciosus	D	May-15	*Malus domestica*	I	Met	La Pintana	-70,634269	-33,568869	623	U	HCOI40	H28S25	19410	INRA	-	KY084975	KY085516
Diaspidiotus perniciosus	D	May-15	*Prunus domestica*	I	Met	Paine	-70,803033	-33,831142	387	C	HCOI40	H28S25	19665	INRA	-	KY085194	KY085515

Species name	Family	Sample Date	Host plant	Type	Region	Municipality	Longitude	Latitude	Altitude (m.)	Area	Haplotype COI	Haplotype 28S	Molecular Code	Voucher Location	Voucher Code	GenBank access. COI	GenBank access. 28S
Diaspidiotus perniciosus	D	May-15	*Prunus domestica*	I	Met	Paine	-70,803033	-33,831142	387	C	HCOI40	H28S25	19666	INRA	-	KY085195	KY085525
Diaspidiotus perniciosus	D	May-15	*Prunus amygdalus*	I	Met	Paine	-70,802383	-33,832083	368	C	HCOI40	-	24761	INRA	-	KY085388	-
Diaspidiotus perniciosus	D	May-15	*Prunus amygdalus*	I	Met	Paine	-70,802383	-33,832083	368	C	-	H28S25	23919	INRA	-	-	KY085505
Diaspidiotus perniciosus	D	May-15	*Prunus amygdalus*	I	Met	Paine	-70,802383	-33,832083	368	C	-	H28S25	23920	INRA	-	-	KY085504
Diaspidiotus perniciosus	D	May-15	*Prunus amygdalus*	I	Met	Paine	-70,802383	-33,832083	368	C	-	H28S25	27925	INRA	-	-	KY085502
Diaspidiotus perniciosus	D	May-15	*Prunus amygdalus*	I	Met	Paine	-70,802383	-33,832083	368	C	-	H28S25	27926	INRA	-	-	KY085501
Diaspidiotus perniciosus	D	May-15	*Cydonia oblonga*	I	Met	Paine	-70,796336	-33,833192	372	C	HCOI40	-	19421	MNHN	1600217	KY084985	-
Diaspidiotus perniciosus	D	May-15	*Cydonia oblonga*	I	Met	Paine	-70,796336	-33,833192	372	C	HCOI40	H28S25	19420	MNHN	1600217	KY084984	KY085512
Diaspidiotus perniciosus	D	Jan-15	*Malus domestica*	I	VI	Placilla	-71,120072	-34,621061	258	C	HCOI40	H28S25	19505	INRA	-	KY085053	KY085521
Diaspidiotus perniciosus	D	Jan-15	*Malus domestica*	I	VI	Placilla	-71,120072	-34,621061	258	C	HCOI40	H28S25	19504	INRA	-	KY085052	KY085523
Diaspidiotus perniciosus	D	Mar-15	*Malus domestica*	I	VI	Placilla	-71,126300	-34,625331	254	C	HCOI40	H28S25	19483	INRA	-	KY085037	KY085518
Diaspidiotus perniciosus	D	Mar-15	*Malus domestica*	I	VI	Placilla	-71,126300	-34,625331	254	C	HCOI40	H28S25	19482	INRA	-	KY085036	KY085522
Diaspis chilensis	D	Apr-15	*Quillaja saponaria*	N1	V	Hijuelas	-71,063406	-32,862842	475	C	HCOI15	H28S09	19427	INRA	-	KY084991	KY085436
Diaspis chilensis	D	Apr-15	*Quillaja saponaria*	N1	V	Hijuelas	-71,063406	-32,862842	475	C	HCOI14	H28S09	19426	INRA	-	KY084990	KY085435
Diaspis chilensis	D	Mar-15	*Nothofagus* sp.	N2	V	Hijuelas	-71,119928	-32,956936	1660	N	HCOI16	-	19705	INRA	-	KY085234	-
Diaspis chilensis	D	Mar-15	*Nothofagus* sp.	N2	V	Hijuelas	-71,124536	-32,957986	1436	N	HCOI16	H28S09	19519	MNHN	1600207	KY085066	KY085433
Diaspis chilensis	D	Mar-15	*Nothofagus* sp.	N2	V	Hijuelas	-71,124536	-32,957986	1436	N	HCOI16	-	19518	MNHN	1600207	KY085065	-
Diaspis chilensis	D	Mar-15	*Quillaja saponaria*	N1	VI	San Fernando	-70,922308	-34,507467	313	U	HCOI13	H28S09	19501	ANSES	1600210	KY085049	KY085432
Diaspis chilensis	D	Mar-15	*Quillaja saponaria*	N1	VI	San Fernando	-70,922308	-34,507467	313	U	HCOI13	H28S09	19500	ANSES	1600210	KY085048	KY085434
Diaspis echinocacti	D	Feb-15	*Echinopsis chiloensis*	N1	V	Olmue	-71,055278	-32,960556	838	N	HCOI08	H28S11	19389	ANSES	1600299	KY084957	KY085442
Epidiaspis leperii	D	May-15	*Pyrus communis*	I	Met	Santiago	-70,633558	-33,569242	625	U	HCOI01	H28S10	19387	ANSES	1600216	KY084955	KY085438
Epidiaspis leperii	D	May-15	*Pyrus communis*	I	Met	Santiago	-70,633558	-33,569242	625	U	HCOI01	H28S10	19386	ANSES	1600216	KY084954	KY085437
Epidiaspis leperii	D	May-15	*Aristotelia chilensis*	N2	VII	Parral	-71,821003	-36,146725	184	U	HCOI01	H28S10	24173	INRA	-	KY085259	KY085441
Epidiaspis leperii	D	May-15	*Aristotelia chilensis*	N2	VIII	Chillan	-72,099961	-36,595719	129	U	HCOI01	H28S10	19405	MNHN	1600312	KY084970	KY085440
Epidiaspis leperii	D	May-15	*Aristotelia chilensis*	N2	VIII	Chillan	-72,099961	-36,595719	129	U	HCOI01	H28S10	19404	INRA	-	KY084969	KY085439
Furchadaspis zamiae	D	Apr-15	*Schinus latifolius*	N2	V	Quillota	-71,193919	-32,872078	176	C	HCOI11	H28S08	19406	ANSES	1600213	KY084971	KY085431
Furchadaspis zamiae	D	Apr-15	*Schinus latifolius*	N2	V	Quillota	-71,193919	-32,872078	176	C	HCOI12	H28S08	19407	ANSES	1600213	KY084972	KY085430
Hemiberlesia lataniae	D	Feb-16	*Yucca*	I	IV	La Serena	-71,242586	-29,919058	92	C	HCOI20	H28S28	24331	INRA	-	KY085353	KY085531
Hemiberlesia lataniae	D	Feb-16	*Yucca*	I	IV	La Serena	-71,242586	-29,919058	92	C	HCOI20	H28S28	24332	INRA	-	KY085354	KY085535
Hemiberlesia lataniae	D	Jun-15	*Persea americana*	I	IV	Monte Patria	-70,871358	-30,720767	563	C	HCOI20	H28S29	19546	INRA	-	KY085086	KY085550
Hemiberlesia lataniae	D	Jun-15	*Persea americana*	I	IV	Monte Patria	-70,871358	-30,720767	563	C	HCOI20	H28S29	19545	INRA	-	KY085085	KY085549
Hemiberlesia lataniae	D	Jun-15	*Persea americana*	I	IV	Monte Patria	-70,881442	-30,730389	565	C	HCOI20	H28S29	19551	INRA	-	KY085088	KY085556
Hemiberlesia lataniae	D	Jun-15	*Persea americana*	I	IV	Monte Patria	-70,881442	-30,730389	565	C	HCOI20	-	19552	INRA	-	KY085089	-
Hemiberlesia lataniae	D	Jun-15	*Persea americana*	I	IV	Monte Patria	-70,770686	-30,851267	715	C	HCOI20	-	19553	INRA	-	KY085090	-
Hemiberlesia lataniae	D	Jun-15	*Persea americana*	I	IV	Monte Patria	-70,770686	-30,851267	715	C	HCOI20	H28S29	19554	INRA	-	KY085091	KY085561
Hemiberlesia lataniae	D	Jun-15	*Persea americana*	I	IV	Monte Patria	-70,770686	-30,851267	715	C	-	H28S29	24120	INRA	-	-	KY085546
Hemiberlesia lataniae	D	Apr-15	*Persea americana*	I	V	Cabildo	-71,103461	-32,438347	240	C	HCOI20	H28S29	19534	INRA	-	KY085075	KY085557
Hemiberlesia lataniae	D	Apr-15	*Persea americana*	I	V	Cabildo	-71,103461	-32,438347	240	C	HCOI20	H28S29	19535	INRA	-	KY085076	KY085566
Hemiberlesia lataniae	D	Apr-15	*Persea americana*	I	V	Cabildo	-71,097144	-32,439528	173	C	HCOI20	H28S29	19509	INRA	-	KY085057	KY085560
Hemiberlesia lataniae	D	Sep-15	*Persea americana*	I	V	Cabildo	-71,103467	-32,440111	216	C	HCOI20	H28S28	24278	INRA	-	KY085322	KY085544
Hemiberlesia lataniae	D	Sep-15	*Persea americana*	I	V	Cabildo	-71,103467	-32,440111	216	C	-	H28S28	24277	INRA	-	-	KY085542
Hemiberlesia lataniae	D	Apr-15	*Persea americana*	I	V	Cabildo	-71,096714	-32,442833	158	C	HCOI20	H28S29	19527	MNHN	1600211	KY085072	KY085558
Hemiberlesia lataniae	D	Apr-15	*Persea americana*	I	V	Cabildo	-71,096714	-32,442833	158	C	HCOI20	H28S29	19526	MNHN	1600211	KY085071	KY085551
Hemiberlesia lataniae	D	Apr-15	*Persea americana*	I	V	Cabildo	-71,102428	-32,444539	170	C	HCOI20	H28S29	19537	INRA	-	KY085078	KY085570
Hemiberlesia lataniae	D	Apr-15	*Persea americana*	I	V	Cabildo	-71,102428	-32,444539	170	C	HCOI18	H28S31	19536	INRA	-	KY085077	KY085574
Hemiberlesia lataniae	D	Apr-15	*Persea americana*	I	V	Cabildo	-71,093250	-32,446242	159	C	HCOI20	H28S29	19524	INRA	-	KY085069	KY085559
Hemiberlesia lataniae	D	Apr-15	*Ficus carica*	I	V	La Cruz	-71,189511	-32,854739	173	C	HCOI18	H28S31	19452	INRA	-	KY085014	KY085575
Hemiberlesia lataniae	D	Apr-15	*Ficus carica*	I	V	La Cruz	-71,189511	-32,854739	173	C	HCOI18	H28S31	19453	INRA	-	KY085015	KY085572
Hemiberlesia lataniae	D	Apr-15	*Persea americana*	I	V	La Cruz	-71,187814	-32,855442	215	C	HCOI20	H28S29	19471	INRA	-	KY085028	KY085548

Species name	Family	Sample Date	Host plant	Type	Region	Municipality	Longitude	Latitude	Altitude (m.)	Area	Haplotype COI	Haplotype 28S	Molecular Code	Voucher Location	Voucher Code	GenBank access. COI	GenBank access. 28S
Hemiberlesia lataniae	D	Apr-15	*Persea americana*	I	V	La Cruz	-71,187814	-32,855442	215	C	HCOI20	H28S29	19472	INRA	-	KY085029	KY085569
Hemiberlesia lataniae	D	Apr-15	*Persea americana*	I	V	La Cruz	-71,190689	-32,855581	176	C	HCOI20	H28S29	19467	INRA	-	KY085025	KY085545
Hemiberlesia lataniae	D	Apr-15	*Persea americana*	I	V	Hijuelas	-71,064389	-32,856881	370	C	HCOI20	H28S30	19450	INRA	-	KY085012	KY085555
Hemiberlesia lataniae	D	Apr-15	*Persea americana*	I	V	Hijuelas	-71,064389	-32,856881	370	C	HCOI20	H28S29	19451	INRA	-	KY085013	KY085563
Hemiberlesia lataniae	D	Apr-15	*Persea americana*	I	V	La Cruz	-71,188622	-32,858200	186	C	HCOI20	H28S29	19459	INRA	-	KY085021	KY085571
Hemiberlesia lataniae	D	Apr-15	*Persea americana*	I	V	La Cruz	-71,188622	-32,858200	186	C	HCOI20	-	19458	INRA	-	KY085020	-
Hemiberlesia lataniae	D	Apr-15	*Persea americana*	I	V	La Cruz	-71,185983	-32,859544	191	C	HCOI20	H28S29	19470	INRA	-	KY085027	KY085568
Hemiberlesia lataniae	D	Apr-15	*Persea americana*	I	V	La Cruz	-71,185983	-32,859544	191	C	HCOI20	H28S29	19469	INRA	-	KY085026	KY085562
Hemiberlesia lataniae	D	Apr-15	*Persea americana*	I	V	Hijuelas	-71,071578	-32,861789	367	C	HCOI20	H28S30	19439	INRA	-	KY085003	KY085553
Hemiberlesia lataniae	D	Apr-15	*Persea americana*	I	V	Hijuelas	-71,071578	-32,861789	367	C	HCOI20	H28S30	19438	INRA	-	KY085002	KY085554
Hemiberlesia lataniae	D	Sep-15	*Persea americana*	I	V	Quillota	-71,189914	-32,862764	172	C	HCOI20	H28S28	24185	INRA	-	KY085268	KY085537
Hemiberlesia lataniae	D	Sep-15	*Persea americana*	I	V	Quillota	-71,189914	-32,862764	172	C	HCOI20	H28S28	24184	INRA	-	KY085267	KY085533
Hemiberlesia lataniae	D	Sep-15	*Persea americana*	I	V	Quillota	-71,188611	-32,863783	179	C	HCOI20	H28S28	24269	INRA	-	KY085316	KY085532
Hemiberlesia lataniae	D	Sep-15	*Persea americana*	I	V	Quillota	-71,188611	-32,863783	179	C	HCOI20	H28S28	24268	INRA	-	KY085315	KY085541
Hemiberlesia lataniae	D	Apr-15	*Persea americana*	I	V	Hijuelas	-71,067125	-32,865375	471	C	HCOI20	H28S29	19446	INRA	-	KY085008	KY085567
Hemiberlesia lataniae	D	Apr-15	*Persea americana*	I	V	Hijuelas	-71,067125	-32,865375	471	C	HCOI20	H28S29	19447	INRA	-	KY085009	KY085565
Hemiberlesia lataniae	D	Sep-15	*Persea americana*	I	V	Hijuelas	-71,074978	-32,869669	501	C	HCOI20	H28S28	24203	INRA	-	KY085277	KY085534
Hemiberlesia lataniae	D	Sep-15	*Persea americana*	I	V	Hijuelas	-71,074978	-32,869669	501	C	HCOI20	H28S28	24204	INRA	-	KY085278	KY085539
Hemiberlesia lataniae	D	Sep-15	*Persea americana*	I	V	Hijuelas	-71,075922	-32,869758	509	C	HCOI20	H28S28	24201	INRA	-	KY085275	KY085536
Hemiberlesia lataniae	D	Sep-15	*Persea americana*	I	V	Hijuelas	-71,075922	-32,869758	509	C	HCOI20	H28S28	24202	INRA	-	KY085276	KY085540
Hemiberlesia lataniae	D	Apr-15	*Persea americana*	I	V	Quillota	-71,194428	-32,874550	163	C	HCOI20	-	19417	INRA	-	KY084981	-
Hemiberlesia lataniae	D	Apr-15	*Persea americana*	I	V	Quillota	-71,194428	-32,874550	163	C	HCOI20	H28S30	19416	INRA	-	KY084980	KY085552
Hemiberlesia lataniae	D	Apr-15	*Pouteria lucuma*	N2	V	Quillota	-71,191833	-32,875178	170	C	HCOI20	-	19652	INRA	-	KY085184	-
Hemiberlesia lataniae	D	May-15	*Schinus latifolius*	N2	V	Cartagena	-71,419708	-33,482900	218	C	-	H28S29	23898	INRA	-	-	KY085547
Hemiberlesia lataniae	D	Feb-15	*Hedera helix*	I	Met	Macul	-70,609167	-33,497611	577	U	HCOI19	H28S31	19512	INRA	-	KY085059	KY085573
Hemiberlesia lataniae	D	Feb-15	*Hedera helix*	I	Met	Macul	-70,609167	-33,497611	577	U	HCOI19	H28S31	19513	INRA	-	KY085060	KY085576
Hemiberlesia lataniae	D	Jun-15	*Persea americana*	I	V	San Antonio	-71,421114	-33,735939	174	U	HCOI20	H28S28	24166	INRA	-	KY085256	KY085538
Hemiberlesia lataniae	D	Jun-15	*Persea americana*	I	V	San Antonio	-71,421114	-33,735939	174	U	HCOI20	H28S28	24165	INRA	-	KY085255	KY085543
Hemiberlesia lataniae	D	Mar-15	*Schinus latifolius*	N2	VI	San Fernando	-70,922308	-34,507467	313	U	HCOI20	H28S29	19498	INRA	-	KY085047	KY085564
Hemiberlesia palmae	D	Nov-15	*Olea europaea*	I	XV	Arica	-69,952539	-18,582586	834	C	HCOI24	H28S22	24286	ANSES	1600693	KY085326	KY085485
Hemiberlesia rapax I	D	Feb-16	*Olea europaea*	I	III	Huasco	-71,157900	-28,481622	37	C	HCOI21	-	24364	INRA	-	KY085378	-
Hemiberlesia rapax I	D	May-15	*Olea europaea*	I	IV	Coquimbo	-71,336386	-29,955581	6	U	HCOI21	H28S21	19390	INRA	-	KY084958	KY085478
Hemiberlesia rapax I	D	May-15	*Olea europaea*	I	IV	Coquimbo	-71,336386	-29,955581	6	U	HCOI21	H28S21	19422	INRA	-	KY084986	KY085480
Hemiberlesia rapax I	D	Apr-15	*Prosopis sp.*	N2	V	Cabildo	-71,103461	-32,438347	240	C	HCOI21	H28S20	19477	INRA	-	KY085032	KY085474
Hemiberlesia rapax I	D	Apr-15	*Prosopis sp.*	N2	V	Cabildo	-71,103461	-32,438347	240	C	HCOI21	H28S20	19478	INRA	-	KY085033	KY085473
Hemiberlesia rapax I	D	Apr-15	*Citrus sinensis*	I	V	Quillota	-71,194831	-32,874394	160	C	HCOI21	H28S21	19412	INRA	-	KY084977	KY085477
Hemiberlesia rapax I	D	Apr-15	*Citrus sinensis*	I	V	Quillota	-71,194831	-32,874394	160	C	HCOI21	H28S21	19413	INRA	-	KY084978	KY085484
Hemiberlesia rapax I	D	Mar-15	*Laurus nobilis*	I	Met	San Bernando	-70,722472	-33,669439	544	U	HCOI21	H28S21	19491	INRA	-	KY085042	KY085482
Hemiberlesia rapax I	D	Mar-15	*Laurus nobilis*	I	Met	San Bernando	-70,722472	-33,669439	544	U	HCOI21	H28S21	19490	INRA	-	KY085041	KY085479
Hemiberlesia rapax I	D	May-15	*Nerium oleander*	I	VII	Curico	-71,206742	-34,978044	234	U	HCOI21	H28S21	19443	MNHN	1600222	KY085005	KY085483
Hemiberlesia rapax I	D	May-15	*Nerium oleander*	I	VII	Curico	-71,206742	-34,978044	234	U	HCOI21	H28S20	19442	MNHN	1600222	KY085004	KY085472
Hemiberlesia rapax I	D	May-15	*Aristotelia chilensis*	N2	VIII	San Carlos	-71,962550	-36,434606	185	U	HCOI21	H28S21	19432	INRA	-	KY084996	KY085475
Hemiberlesia rapax I	D	May-15	*Laurus nobilis*	I	VIII	Chillan	-72,099961	-36,595719	129	U	HCOI21	H28S21	19415	INRA	-	KY084979	KY085481
Hemiberlesia rapax I	D	May-15	*Laurus nobilis*	I	VIII	Chillan	-72,099961	-36,595719	129	U	-	H28S21	27919	INRA	-	-	KY085476
Hemiberlesia rapax II	D	Nov-15	*Olea europaea*	I	XV	Arica	-69,952539	-18,582586	834	C	-	H28S15	24287	INRA	-	-	KY085464
Hemiberlesia rapax II	D	Jul-15	*Acacia retinodes*	I	I	Pisagua	-70,211583	-19,596589	12	U	-	H28S15	24167	INRA	-	-	KY085463
Hemiberlesia rapax II	D	Feb-16	*Vitis vinifera*	I	IV	Vicuña	-70,696786	-30,035639	632	U	HCOI37	-	24356	ANSES	1600298	KY085373	-
Hemiberlesia rapax II	D	Apr-15	*From rearing*	-	V	Quillota	-71,308356	-32,936886	75	C	HCOI38	-	27906	INRA	-	KY085391	-

Species name	Family	Sample Date	Host plant	Type	Region	Municipality	Longitude	Latitude	Altitude (m.)	Area	Haplotype COI	Haplotype 28S	Molecular Code	Voucher Location	Voucher Code	GenBank access. COI	GenBank access. 28S
Hemiberlesia rapax II	D	May-15	*Schinus latifolius*	N2	V	Casablanca	-71,394678	-33,457094	241	C	HCOI39	-	27915	INRA	-	KY085393	-
Hemiberlesia rapax II	D	May-15	*Schinus latifolius*	N2	V	Casablanca	-71,394678	-33,457094	241	C	HCOI39	-	27914	INRA	-	KY085392	-
Hemiberlesia rapax II	D	Feb-16	*Schinus molle*	N1	VI	Rancagua	-70,740697	-34,155800	494	U	-	H28S15	24380	INRA	-	-	KY085462
Hemiberlesia rapax II	D	Mar-15	*Aristotelia chilensis*	N2	VII	Sagrada Familia	-71,306525	-35,034678	200	C	HCOI38	-	27916	INRA	-	KY085394	-
Hemiberlesia rapax III	D	Feb-16	*Vitis vinifera*	I	IV	Vicuña	-70,696786	-30,035639	632	U	-	H28S16	24355	INRA	-	-	KY085465
Hemiberlesia rapax III	D	Jun-15	*Schinus molle*	N1	IV	Monte Patria	-70,881442	-30,730389	565	C	Pseudogene	H28S19	19547	ANSES	1600302	KY084950	KY085471
Hemiberlesia rapax III	D	Mar-15	Unknow	-	V	Hijuelas	-71,122714	-32,962989	1178	N	Pseudogene	H28S17	19520	INRA	-	KY084952	KY085470
Hemiberlesia rapax III	D	Mar-15	Unknow	-	V	Hijuelas	-71,122714	-32,962989	1178	N	Pseudogene	H28S18	19521	MNHN	1600208	KY084953	KY085469
Hemiberlesia rapax III	D	May-15	*Schinus latifolius*	N2	V	Casablanca	-71,394678	-33,457094	241	C	Pseudogene	H28S17	19440	ANSES	1600304	KY084948	KY085467
Hemiberlesia rapax III	D	Mar-15	*Aristotelia chilensis*	N2	VII	Sagrada Familia	-71,306525	-35,034678	200	C	Pseudogene	H28S17	19492	ANSES	1600303	KY084951	KY085466
Hemiberlesia rapax III	D	May-15	*Laurus nobilis*	I	VIII	Chillan	-72,099961	-36,595719	129	U	Pseudogene	H28S17	19414	INRA	-	KY084949	KY085468
Lepidosaphes beckii	D	Feb-16	*Citrus sinensis*	I	III	Vallenar	-70,797103	-28,581025	470	C	HCOI05	H28S01	24366	INRA	-	KY085379	KY085412
Lepidosaphes beckii	D	Feb-16	*Citrus sinensis*	I	III	Vallenar	-70,797103	-28,581025	470	C	-	H28S01	24367	INRA	-	-	KY085401
Lepidosaphes beckii	D	Feb-16	*Citrus sinensis*	I	IV	Coquimbo	-71,250056	-29,984811	120	C	HCOI03	H28S01	24374	INRA	-	KY085383	KY085413
Lepidosaphes beckii	D	Feb-16	*Citrus sinensis*	I	IV	Coquimbo	-71,250056	-29,984811	120	C	HCOI05	H28S01	24375	INRA	-	KY085384	KY085411
Lepidosaphes beckii	D	May-15	*Citrus sp.*	I	IV	Vicuña	-70,819556	-30,051969	589	N	HCOI02	H28S01	19388	INRA	-	KY084956	KY085397
Lepidosaphes beckii	D	Jun-15	*Citrus limon*	I	IV	Ovalle	-71,144531	-30,537914	286	C	HCOI05	H28S01	19567	INRA	-	KY085104	KY085400
Lepidosaphes beckii	D	Jun-15	*Citrus limon*	I	IV	Ovalle	-71,144531	-30,537914	286	C	HCOI05	H28S01	19568	INRA	-	KY085105	KY085410
Lepidosaphes beckii	D	Sep-15	*Citrus limon*	I	V	Cabildo	-70,934694	-32,486486	333	U	HCOI05	H28S01	24239	ANSES	1600295	KY085294	KY085403
Lepidosaphes beckii	D	Sep-15	*Citrus limon*	I	V	Cabildo	-70,934694	-32,486486	333	U	HCOI05	H28S01	24238	ANSES	1600295	KY085293	KY085408
Lepidosaphes beckii	D	Jan-16	*Citrus sinensis*	I	V	Putaendo	-70,716858	-32,622711	831	U	HCOI05	H28S01	24330	INRA	-	KY085352	KY085404
Lepidosaphes beckii	D	Jan-16	*Citrus sinensis*	I	V	Putaendo	-70,716858	-32,622711	831	U	HCOI05	H28S01	24329	INRA	-	KY085351	KY085398
Lepidosaphes beckii	D	Apr-15	*Citrus aurantifolia*	I	V	Quillota	-71,232747	-32,848558	158	U	HCOI05	H28S01	19436	INRA	-	KY085000	KY085405
Lepidosaphes beckii	D	Apr-15	*Citrus aurantifolia*	I	V	Quillota	-71,232747	-32,848558	158	U	HCOI04	H28S01	19437	INRA	-	KY085001	KY085395
Lepidosaphes beckii	D	Sep-15	*Citrus sinensis*	I	V	Quillota	-71,192083	-32,871936	174	C	HCOI05	H28S01	24270	INRA	-	KY085317	KY085406
Lepidosaphes beckii	D	Sep-15	*Citrus sinensis*	I	V	Quillota	-71,192083	-32,871936	174	C	HCOI05	H28S01	24271	INRA	-	KY085318	KY085402
Lepidosaphes beckii	D	Apr-15	*Citrus sinensis*	I	V	Quillota	-71,195847	-32,873494	158	C	HCOI03	H28S01	19543	INRA	-	KY085083	KY085409
Lepidosaphes beckii	D	Feb-15	*Citrus limon*	I	V	Valparaiso	-71,630556	-33,047500	114	U	HCOI05	H28S02	19510	INRA	-	KY085058	KY085396
Lepidosaphes beckii	D	Mar-15	*Citrus sinensis*	I	VI	Placilla	-71,124206	-34,625253	254	C	HCOI05	H28S01	19495	INRA	-	KY085044	KY085407
Lepidosaphes beckii	D	Mar-15	*Citrus sinensis*	I	VI	Placilla	-71,124206	-34,625253	254	C	HCOI05	H28S01	19494	INRA	-	KY085043	KY085399
Lepidosaphes ulmi	D	Jun-15	*Prunus persica*	I	IV	Ovalle	-71,144531	-30,537914	286	C	HCOI06	H28S03	19557	MNHN	1600228	KY085094	KY085419
Lepidosaphes ulmi	D	Jun-15	*Prunus persica*	I	IV	Ovalle	-71,144531	-30,537914	286	C	HCOI06	-	19558	MNHN	1600228	KY085095	-
Lepidosaphes ulmi	D	Jun-15	*Persea americana*	I	IV	Monte Patria	-70,770686	-30,851267	715	C	HCOI07	H28S03	19549	INRA	-	KY085087	KY085418
Lepidosaphes ulmi	D	Apr-15	*Prosopis sp.*	N2	V	Cabildo	-71,103461	-32,438347	240	C	-	H28S04	19475	INRA	-	-	KY085420
Lepidosaphes ulmi	D	Apr-15	*Prosopis sp.*	N2	V	Cabildo	-71,103461	-32,438347	240	C	-	H28S03	19476	INRA	-	-	KY085415
Lepidosaphes ulmi	D	Mar-15	*Pyrus communis*	I	Met	La Pintana	-70,633558	-33,569242	625	U	-	H28S03	19531	INRA	-	-	KY085414
Lepidosaphes ulmi	D	Mar-15	Unknow	-	Met	Nueva Independencia	-70,762842	-33,643031	510	U	-	H28S03	19532	INRA	-	-	KY085416
Lepidosaphes ulmi	D	Mar-15	*Juglans regia*	I	Met	Paine	-70,796772	-33,834250	369	C	HCOI07	-	19530	INRA	-	KY085073	-
Lepidosaphes ulmi	D	Mar-15	*Juglans regia*	I	Met	Paine	-70,796772	-33,834250	369	C	-	H28S03	19529	INRA	-	-	KY085417
Melanaspis sitreana	D	Mar-15	*Nothofagus*	N2	V	Hijuelas	-71,119928	-32,956936	1660	N	HCOI27	H28S12	19522	MNHN	1600209	KY085067	KY085444
Melanaspis sitreana	D	Mar-15	*Nothofagus*	N2	V	Hijuelas	-71,119928	-32,956936	1660	N	HCOI27	H28S12	19523	MNHN	1600209	KY085068	KY085445
Melanaspis sitreana	D	May-15	*Quillaja saponaria*	N1	V	Casablanca	-71,312872	-33,364111	313	C	-	H28S12	19613	INRA	-	-	KY085443
Melanaspis sitreana	D	May-15	*Quillaja saponaria*	N1	V	Casablanca	-71,312872	-33,364111	313	C	-	H28S12	19614	INRA	-	-	KY085446
Parasaissetia nigra	C	Jun-15	*Annona cherimola*	N2	IV	Ovalle	-71,144531	-30,537914	286	C	HCOI56	H28S50	19722	ANSES	1600227	KY085249	KY085828
Parasaissetia nigra	C	Jun-15	*Annona cherimola*	N2	IV	Ovalle	-71,144531	-30,537914	286	C	HCOI56	H28S50	19721	ANSES	1600227	KY085248	KY085827
Parthenolecanium corni	C	Sep-15	Unknow	-	V	Cabildo	-71,098039	-32,442889	158	C	HCOI42	H28S52	24244	INRA	-	KY085296	KY085850
Parthenolecanium corni	C	Sep-15	Unknow	-	V	Cabildo	-71,098039	-32,442889	158	C	HCOI42	H28S52	24245	INRA	-	KY085297	KY085852
Parthenolecanium corni	C	Sep-15	*Prunus persica*	I	V	Hijuelas	-71,069469	-32,859256	351	C	HCOI44	H28S52	24216	INRA	-	KY085284	KY085851

Species name	Family	Sample Date	Host plant	Type	Region	Municipality	Longitude	Latitude	Altitude (m.)	Area	Haplotype COI	Haplotype 28S	Molecular Code	Voucher Location	Voucher Code	GenBank access. COI	GenBank access. 28S
Parthenolecanium corni	C	Sep-15	*Prunus persica*	I	V	Hijuelas	-71,069469	-32,859256	351	C	HCOI44	H28S52	24215	ANSES	1600290	KY085283	KY085849
Parthenolecanium corni	C	May-15	*Aristotelia chilensis*	N2	VII	Parral	-71,821003	-36,146725	184	U	HCOI42	H28S52	19597	ANSES	1600224	KY085133	KY085848
Parthenolecanium corni	C	May-15	*Aristotelia chilensis*	N2	VII	Parral	-71,821003	-36,146725	184	U	HCOI43	H28S52	19596	ANSES	1600224	KY085132	KY085847
Protopulvinaria pyriformis	C	May-15	*Schefflera arboricola*	I	IV	La Serena	-71,246031	-29,901497	34	U	HCOI54	H28S54	19588	INRA	-	KY085124	KY085878
Protopulvinaria pyriformis	C	May-15	*Hedera helix*	I	IV	La Serena	-71,245303	-29,916150	47	C	HCOI54	H28S54	19631	ANSES	1600300	KY085163	KY085874
Protopulvinaria pyriformis	C	May-15	*Hedera helix*	I	IV	La Serena	-71,245303	-29,916150	47	C	HCOI54	H28S54	19630	INRA	-	KY085162	KY085879
Protopulvinaria pyriformis	C	Feb-16	*Hedera helix*	I	IV	La Serena	-71,242586	-29,919058	92	C	HCOI54	H28S53	24341	INRA	-	KY085362	KY085863
Protopulvinaria pyriformis	C	Feb-16	*Hedera helix*	I	IV	La Serena	-71,242586	-29,919058	92	C	HCOI54	-	24342	INRA	-	KY085363	-
Protopulvinaria pyriformis	C	Apr-15	*Cryptocarya alba*	N1	V	La Cruz	-71,199806	-32,854186	146	C	HCOI54	H28S54	19701	INRA	-	KY085230	KY085882
Protopulvinaria pyriformis	C	Apr-15	*Cryptocarya alba*	N1	V	La Cruz	-71,199806	-32,854186	146	C	HCOI54	H28S54	19702	INRA	-	KY085231	KY085875
Protopulvinaria pyriformis	C	Feb-15	*Persea americana*	I	V	La Cruz	-71,187814	-32,855442	215	C	HCOI54	H28S54	19430	INRA	-	KY084994	KY085872
Protopulvinaria pyriformis	C	Feb-15	*Persea americana*	I	V	La Cruz	-71,187814	-32,855442	215	C	HCOI54	H28S54	19431	INRA	-	KY084995	KY085866
Protopulvinaria pyriformis	C	Apr-15	*Persea americana*	I	V	La Cruz	-71,188622	-32,858200	186	C	HCOI54	H28S54	19691	INRA	-	KY085220	KY085868
Protopulvinaria pyriformis	C	Apr-15	*Persea americana*	I	V	La Cruz	-71,188622	-32,858200	186	C	HCOI54	H28S54	19692	INRA	-	KY085221	KY085869
Protopulvinaria pyriformis	C	Sep-15	*Persea americana*	I	V	Quillota	-71,189922	-32,862494	172	C	HCOI54	H28S53	24178	INRA	-	KY085263	KY085853
Protopulvinaria pyriformis	C	Sep-15	*Persea americana*	I	V	Quillota	-71,189922	-32,862494	172	C	HCOI54	H28S53	24179	INRA	-	KY085264	KY085857
Protopulvinaria pyriformis	C	Sep-15	*Persea americana*	I	V	Quillota	-71,184086	-32,863000	201	C	HCOI54	H28S53	24261	INRA	-	KY085308	KY085864
Protopulvinaria pyriformis	C	Sep-15	*Persea americana*	I	V	Quillota	-71,184086	-32,863000	201	C	HCOI54	H28S53	24262	INRA	-	KY085309	KY085862
Protopulvinaria pyriformis	C	Apr-15	*Cryptocarya alba*	N1	V	Hijuelas	-71,067125	-32,865375	471	C	HCOI54	H28S54	19679	INRA	-	KY085208	KY085870
Protopulvinaria pyriformis	C	Apr-15	*Cryptocarya alba*	N1	V	Hijuelas	-71,067125	-32,865375	471	C	HCOI54	H28S54	19680	INRA	-	KY085209	KY085867
Protopulvinaria pyriformis	C	Sep-15	*Quillaja saponaria*	N1	V	Hijuelas	-71,075936	-32,866392	410	C	HCOI54	H28S53	24194	INRA	-	KY085272	KY085854
Protopulvinaria pyriformis	C	Sep-15	*Quillaja saponaria*	N1	V	Hijuelas	-71,075936	-32,866392	410	C	HCOI54	H28S53	24195	INRA	-	KY085273	KY085858
Protopulvinaria pyriformis	C	Sep-15	*Hedera helix*	I	V	Quillota	-71,192197	-32,875308	167	C	HCOI54	H28S53	24264	INRA	-	KY085311	KY085856
Protopulvinaria pyriformis	C	Sep-15	*Hedera helix*	I	V	Quillota	-71,192197	-32,875308	167	C	HCOI54	H28S53	24265	INRA	-	KY085312	KY085855
Protopulvinaria pyriformis	C	May-15	*Laurus nobilis*	I	V	Quillota	-71,244819	-32,878986	135	U	HCOI54	H28S54	19599	INRA	-	KY085135	KY085880
Protopulvinaria pyriformis	C	May-15	*Laurus nobilis*	I	V	Quillota	-71,244819	-32,878986	135	U	HCOI54	H28S54	19598	INRA	-	KY085134	KY085871
Protopulvinaria pyriformis	C	Apr-15	*Hedera helix*	I	V	Quillota	-71,209356	-32,896217	140	U	HCOI54	H28S54	19689	INRA	-	KY085218	KY085876
Protopulvinaria pyriformis	C	Apr-15	*Hedera helix*	I	V	Quillota	-71,209356	-32,896217	140	U	HCOI54	H28S54	19690	INRA	-	KY085219	KY085877
Protopulvinaria pyriformis	C	Apr-15	*Hedera helix*	I	V	Valparaiso	-71,626642	-33,041225	36	U	HCOI54	H28S54	19651	INRA	-	KY085183	KY085873
Protopulvinaria pyriformis	C	Apr-15	*Hedera helix*	I	V	Valparaiso	-71,626642	-33,041225	36	U	HCOI54	H28S54	19650	INRA	-	KY085182	KY085881
Protopulvinaria pyriformis	C	Jan-16	*Hedera helix*	I	Met	Paine	-70,755939	-33,855500	378	U	HCOI54	H28S53	24321	INRA	-	KY085348	KY085865
Protopulvinaria pyriformis	C	Jan-16	*Hedera helix*	I	Met	Paine	-70,755939	-33,855500	378	U	HCOI54	H28S53	24322	INRA	-	KY085349	KY085860
Protopulvinaria pyriformis	C	Oct-15	*Hedera helix*	I	VI	Rancagua	-70,733764	-34,167022	508	U	HCOI54	H28S53	24260	INRA	-	KY085307	KY085861
Protopulvinaria pyriformis	C	Oct-15	*Hedera helix*	I	VI	Rancagua	-70,733764	-34,167022	508	U	HCOI54	H28S53	24259	INRA	-	KY085306	KY085859
Pseudoparlatoria chilina	D	Nov-15	*Laureliopsis philippiana*	N2	XIV	Puyehue	-72,172134	-40,668603	361	N	HCOI17	H28S05	24295	ANSES	1600296	KY085330	KY085422
Pseudoparlatoria chilina	D	Nov-15	*Laureliopsis philippiana*	N2	XIV	Puyehue	-72,172134	-40,668603	361	N	HCOI17	H28S05	24294	ANSES	1600296	KY085329	KY085421
Pseudoparlatoria sp.	D	Nov-15	*Aextoxicon punctatum*	N2	XIV	Puyehue	-72,172134	-40,668603	361	N	-	H28S06	24298	INRA	-	-	KY085424
Pseudoparlatoria sp.	D	Nov-15	Myrtaceae	N2	XIV	Puyehue	-72,172134	-40,668603	361	N	-	H28S06	24290	ANSES	1600301	-	KY085423
Pseudoparlatoria sp.	D	Nov-15	Myrtaceae	N2	XIV	Puyehue	-72,172134	-40,668603	361	N	-	H28S06	24291	ANSES	1600301	-	KY085425
Pulvinariella mesembryanthemi	C	Nov-15	*Ilex sp.*	I	XIV	Valdivia	-73,249583	-39,804917	15	U	HCOI55	H28S55	24308	INRA	-	KY085339	KY085885
Pulvinariella mesembryanthemi	C	Nov-15	*Ilex sp.*	I	XIV	Valdivia	-73,249583	-39,804917	15	U	-	H28S55	24307	INRA	-	-	KY085888
Pulvinariella mesembryanthemi	C	Nov-15	*Choisya ternata*	I	XIV	Valdivia	-73,251028	-39,806264	18	U	HCOI55	H28S55	24300	INRA	-	KY085334	KY085884
Pulvinariella mesembryanthemi	C	Nov-15	*Choisya ternata*	I	XIV	Valdivia	-73,251028	-39,806264	18	U	HCOI55	H28S55	24299	INRA	-	KY085333	KY085886
Pulvinariella mesembryanthemi	C	Nov-15	*Hedera helix*	I	X	Osorno	-72,981211	-40,598800	91	U	HCOI55	H28S55	24297	INRA	-	KY085332	KY085887
Pulvinariella mesembryanthemi	C	Nov-15	*Hedera helix*	I	X	Osorno	-72,981211	-40,598800	91	U	HCOI55	H28S55	24296	INRA	-	KY085331	KY085889
Saissetia coffeae	C	Nov-15	*Olea europaea*	I	XV	Arica	-69,952539	-18,582586	834	C	-	H28S45	24289	INRA	-	-	KY085809
Saissetia coffeae	C	Nov-15	*Olea europaea*	I	XV	Arica	-69,952539	-18,582586	834	C	-	H28S45	24305	INRA	-	-	KY085808
Saissetia coffeae	C	Nov-15	*Olea europaea*	I	XV	Arica	-69,952539	-18,582586	834	C	-	H28S45	24306	INRA	-	-	KY085811
Saissetia coffeae	C	Nov-15	*Olea europaea*	I	XV	Arica	-69,952539	-18,582586	834	C	-	H28S45	24288	INRA	-	-	KY085810

Species name	Family	Sample Date	Host plant	Type	Region	Municipality	Longitude	Latitude	Altitude (m.)	Area	Haplotype COI	Haplotype 28S	Molecular Code	Voucher Location	Voucher Code	GenBank access. COI	GenBank access. 28S
Saissetia coffeae	C	Jul-15	Unknow	-	I	Iquique	-70,150564	-20,217608	13	U	-	H28S45	24158	INRA	-	-	KY085812
Saissetia coffeae	C	Feb-16	*Olea europaea*	I	III	Vallenar	-70,795058	-28,578758	452	C	HCOI57	H28S46	24378	INRA	-	KY085387	KY085818
Saissetia coffeae	C	Feb-16	*Olea europaea*	I	III	Vallenar	-70,795058	-28,578758	452	C	-	H28S46	24379	INRA	-	-	KY085817
Saissetia coffeae	C	May-15	*Olea europaea*	I	IV	La Serena	-71,147150	-29,939536	120	U	HCOI57	H28S46	19624	MNHN	1600218	KY085156	KY085816
Saissetia coffeae	C	May-15	*Olea europaea*	I	IV	Coquimbo	-71,336386	-29,955581	6	U	HCOI57	H28S46	19619	MNHN	1600220	KY085151	KY085813
Saissetia coffeae	C	May-15	*Olea europaea*	I	IV	Coquimbo	-71,336386	-29,955581	6	U	HCOI57	H28S46	19620	MNHN	1600220	KY085152	KY085815
Saissetia oleae	C	Jul-15	*Nerium oleander*	I	I	Pisagua	-70,212242	-19,596919	11	U	-	H28S38	24160	INRA	-	-	KY085719
Saissetia oleae	C	Jul-15	*Nerium oleander*	I	I	Pisagua	-70,212242	-19,596919	11	U	-	H28S39	24159	INRA	-	-	KY085750
Saissetia oleae	C	Feb-16	*Citrus sinensis*	I	III	Vallenar	-70,797103	-28,581025	470	C	-	H28S43	24347	INRA	-	-	KY085800
Saissetia oleae	C	Feb-16	*Citrus sinensis*	I	IV	La Serena	-71,242586	-29,919058	92	C	-	H28S43	24340	INRA	-	-	KY085797
Saissetia oleae	C	Feb-16	*Citrus sinensis*	I	IV	Coquimbo	-71,250056	-29,984811	120	C	HCOI60	H28S38	24372	INRA	-	KY085382	KY085720
Saissetia oleae	C	Sep-15	*Olea europaea*	I	V	La Ligua	-71,295286	-32,321933	74	U	-	H28S43	24205	INRA	-	-	KY085782
Saissetia oleae	C	Sep-15	*Olea europaea*	I	V	La Ligua	-71,295286	-32,321933	74	U	-	H28S43	24206	INRA	-	-	KY085787
Saissetia oleae	C	Apr-15	*Persea americana*	I	V	Cabildo	-71,103461	-32,438347	240	C	HCOI60	-	19714	INRA	-	KY085241	-
Saissetia oleae	C	Apr-15	*Persea americana*	I	V	Cabildo	-71,097144	-32,439528	173	C	HCOI61	H28S36	19712	INRA	-	KY085239	KY085673
Saissetia oleae	C	Apr-15	*Persea americana*	I	V	Cabildo	-71,097144	-32,439528	173	C	HCOI61	-	19713	INRA	-	KY085240	-
Saissetia oleae	C	Sep-15	*Prunus persica*	I	V	Cabildo	-71,103022	-32,439975	216	C	-	H28S43	24236	INRA	-	-	KY085779
Saissetia oleae	C	Apr-15	*Persea americana*	I	V	Cabildo	-71,100917	-32,442600	172	C	HCOI61	-	19715	INRA	-	KY085242	-
Saissetia oleae	C	Apr-15	*Persea americana*	I	V	Cabildo	-71,100917	-32,442600	172	C	HCOI61	-	19716	INRA	-	KY085243	-
Saissetia oleae	C	Sep-15	*Olea europaea*	I	V	Cabildo	-71,118781	-32,473328	133	U	-	H28S43	24240	INRA	-	-	KY085785
Saissetia oleae	C	Sep-15	*Olea europaea*	I	V	Cabildo	-71,118781	-32,473328	133	U	-	H28S43	24241	INRA	-	-	KY085793
Saissetia oleae	C	Sep-15	*Olea europaea*	I	V	Cabildo	-70,961386	-32,494317	327	U	-	H28S38	24234	INRA	-	-	KY085726
Saissetia oleae	C	Sep-15	*Olea europaea*	I	V	Cabildo	-70,961386	-32,494317	327	U	-	H28S38	24235	INRA	-	-	KY085728
Saissetia oleae	C	Sep-15	*Citrus clementina*	I	V	Putaendo	-70,716706	-32,622722	831	U	-	H28S42	24229	INRA	-	-	KY085757
Saissetia oleae	C	Sep-15	*Olea europaea*	I	V	Putaendo	-70,719083	-32,640931	790	U	-	H28S43	24230	INRA	-	-	KY085795
Saissetia oleae	C	Sep-15	*Olea europaea*	I	V	Putaendo	-70,719083	-32,640931	790	U	-	H28S43	24231	INRA	-	-	KY085801
Saissetia oleae	C	Sep-15	*Nerium oleander*	I	V	Catemu	-70,869825	-32,791336	499	U	HCOI61	H28S38	24220	INRA	-	KY085285	KY085727
Saissetia oleae	C	Sep-15	*Nerium oleander*	I	V	Catemu	-70,869825	-32,791336	499	U	-	H28S38	24219	INRA	-	-	KY085721
Saissetia oleae	C	Aug-15	*Schinus molle*	N1	V	Hijuelas	-71,046436	-32,850111	438	C	-	H28S38	24176	INRA	-	-	KY085724
Saissetia oleae	C	Aug-15	*Schinus molle*	N1	V	Hijuelas	-71,046436	-32,850111	438	C	HCOI61	H28S43	24177	INRA	-	KY085262	KY085796
Saissetia oleae	C	Apr-15	*Persea americana*	I	V	La Cruz	-71,184600	-32,854442	214	C	HCOI61	H28S38	19693	INRA	-	KY085222	KY085687
Saissetia oleae	C	Apr-15	*Persea americana*	I	V	La Cruz	-71,184600	-32,854442	214	C	HCOI61	-	19694	INRA	-	KY085223	-
Saissetia oleae	C	Apr-15	*Persea americana*	I	V	La Cruz	-71,187814	-32,855442	215	C	HCOI61	H28S36	19717	INRA	-	KY085244	KY085670
Saissetia oleae	C	Apr-15	*Persea americana*	I	V	La Cruz	-71,187814	-32,855442	215	C	HCOI61	H28S37	19718	INRA	-	KY085245	KY085679
Saissetia oleae	C	Apr-15	*Olea europaea*	I	V	La Cruz	-71,190689	-32,855581	176	C	HCOI61	H28S38	19720	INRA	-	KY085247	KY085710
Saissetia oleae	C	Apr-15	*Olea europaea*	I	V	La Cruz	-71,190689	-32,855581	176	C	HCOI61	-	19719	INRA	-	KY085246	-
Saissetia oleae	C	Apr-15	Unknow	-	V	Hijuelas	-71,064389	-32,856881	370	C	HCOI60	H28S38	19585	INRA	-	KY085121	KY085706
Saissetia oleae	C	Apr-15	*Persea americana*	I	V	Hijuelas	-71,064389	-32,856881	370	C	HCOI61	H28S38	19626	INRA	-	KY085158	KY085702
Saissetia oleae	C	Apr-15	*Prosopis sp.*	N2	V	Hijuelas	-71,064389	-32,856881	370	C	HCOI61	H28S38	19638	INRA	-	KY085170	KY085700
Saissetia oleae	C	Apr-15	*Persea americana*	I	V	Hijuelas	-71,064389	-32,856881	370	C	HCOI61	H28S43	19627	INRA	-	KY085159	KY085774
Saissetia oleae	C	Apr-15	*Prosopis sp.*	N2	V	Hijuelas	-71,064389	-32,856881	370	C	HCOI61	H28S44	19639	INRA	-	KY085171	KY085804
Saissetia oleae	C	Apr-15	Unknow	-	V	Hijuelas	-71,064389	-32,856881	370	C	HCOI61	-	19584	INRA	-	KY085120	-
Saissetia oleae	C	Feb-15	*Persea americana*	I	V	Hijuelas	-71,066550	-32,857528	351	C	HCOI61	H28S38	19573	MNHN	1600205	KY085109	KY085685
Saissetia oleae	C	Feb-15	*Persea americana*	I	V	Hijuelas	-71,066550	-32,857528	351	C	HCOI61	H28S43	19571	INRA	-	KY085107	KY085765
Saissetia oleae	C	Feb-15	*Persea americana*	I	V	Hijuelas	-71,066550	-32,857528	351	C	HCOI61	H28S43	19570	INRA	-	KY085106	KY085761
Saissetia oleae	C	Feb-15	*Persea americana*	I	V	Hijuelas	-71,066550	-32,857528	351	C	HCOI61	H28S44	19572	MNHN	1600205	KY085108	KY085805
Saissetia oleae	C	Sep-15	Unknow	-	V	Hijuelas	-71,063342	-32,858589	392	C	HCOI60	H28S38	24272	INRA	-	KY085319	KY085729
Saissetia oleae	C	Sep-15	*Persea americana*	I	V	Hijuelas	-71,079886	-32,860136	329	C	HCOI61	H28S43	24186	INRA	-	KY085269	KY085790
Saissetia oleae	C	Sep-15	*Persea americana*	I	V	Hijuelas	-71,079886	-32,860136	329	C	-	H28S43	24187	INRA	-	-	KY085788

Species name	Family	Sample Date	Host plant	Type	Region	Municipality	Longitude	Latitude	Altitude (m.)	Area	Haplotype COI	Haplotype 28S	Molecular Code	Voucher Location	Voucher Code	GenBank access. COI	GenBank access. 28S
Saissetia oleae	C	Apr-15	*Persea americana*	I	V	Hijuelas	-71,071578	-32,861789	367	C	HCOI60	H28S38	19602	INRA	-	KY085137	KY085701
Saissetia oleae	C	Apr-15	*Persea americana*	I	V	Hijuelas	-71,071578	-32,861789	367	C	HCOI61	H28S43	19601	INRA	-	KY085136	KY085762
Saissetia oleae	C	Sep-15	*Citrus limon*	I	V	Hijuelas	-71,065958	-32,862044	425	C	HCOI61	H28S41	24221	INRA	-	KY085286	KY085755
Saissetia oleae	C	Sep-15	*Quillaja saponaria*	N1	V	Quillota	-71,186422	-32,865106	185	C	-	H28S43	24182	INRA	-	-	KY085786
Saissetia oleae	C	Apr-15	*Schinus latifolius*	N2	V	Hijuelas	-71,067125	-32,865375	471	C	HCOI61	H28S37	19667	INRA	-	KY085196	KY085674
Saissetia oleae	C	Apr-15	*Schinus latifolius*	N2	V	Hijuelas	-71,067125	-32,865375	471	C	HCOI61	H28S39	19668	INRA	-	KY085197	KY085735
Saissetia oleae	C	Apr-15	*Persea americana*	I	V	Hijuelas	-71,067125	-32,865375	471	C	HCOI61	H28S43	19675	INRA	-	KY085204	KY085766
Saissetia oleae	C	Apr-15	*Persea americana*	I	V	Hijuelas	-71,067125	-32,865375	471	C	HCOI62	-	19676	INRA	-	KY085205	-
Saissetia oleae	C	Sep-15	*Quillaja saponaria*	N1	V	Hijuelas	-71,075936	-32,866392	410	C	-	H28S43	24193	INRA	-	-	KY085784
Saissetia oleae	C	Sep-15	*Quillaja saponaria*	N1	V	Hijuelas	-71,075936	-32,866392	410	C	-	H28S43	24192	INRA	-	-	KY085780
Saissetia oleae	C	Sep-15	*Persea americana*	I	V	Hijuelas	-71,074164	-32,866486	423	C	-	H28S43	24197	INRA	-	-	KY085794
Saissetia oleae	C	Sep-15	*Persea americana*	I	V	Hijuelas	-71,074164	-32,866486	423	C	-	H28S43	24198	INRA	-	-	KY085789
Saissetia oleae	C	Sep-15	*Persea americana*	I	V	Hijuelas	-71,079108	-32,868742	450	C	-	H28S38	24200	INRA	-	-	KY085722
Saissetia oleae	C	Sep-15	*Persea americana*	I	V	Hijuelas	-71,079108	-32,868742	450	C	-	H28S43	24199	INRA	-	-	KY085791
Saissetia oleae	C	Apr-15	*Schinus latifolius*	N2	V	Quillota	-71,193919	-32,872078	176	C	HCOI61	H28S44	19622	INRA	-	KY085154	KY085803
Saissetia oleae	C	Apr-15	*Schinus latifolius*	N2	V	Quillota	-71,193919	-32,872078	176	C	HCOI61	H28S44	19623	INRA	-	KY085155	KY085806
Saissetia oleae	C	Apr-15	*Maytenus boaria*	N2	V	Quillota	-71,194192	-32,872092	165	C	HCOI60	H28S44	19658	INRA	-	KY085189	KY085807
Saissetia oleae	C	Apr-15	*Maytenus boaria*	N2	V	Quillota	-71,194192	-32,872092	165	C	HCOI60	-	19659	INRA	-	KY085190	-
Saissetia oleae	C	Apr-15	*Olea europaea*	I	V	Quillota	-71,195742	-32,872108	159	C	HCOI60	H28S38	19629	INRA	-	KY085161	KY085690
Saissetia oleae	C	Apr-15	*Tristerix tetrandrus*	N2	V	Quillota	-71,195742	-32,872108	159	C	HCOI61	H28S38	19634	INRA	-	KY085166	KY085693
Saissetia oleae	C	Apr-15	*Olea europaea*	I	V	Quillota	-71,195742	-32,872108	159	C	HCOI61	H28S39	19628	INRA	-	KY085160	KY085734
Saissetia oleae	C	Apr-15	*Tristerix tetrandrus*	N2	V	Quillota	-71,195742	-32,872108	159	C	HCOI60	H28S43	19635	INRA	-	KY085167	KY085764
Saissetia oleae	C	Apr-15	*Citrus sinensis*	I	V	Quillota	-71,194653	-32,872836	165	C	HCOI60	H28S43	19632	INRA	-	KY085164	KY085771
Saissetia oleae	C	Apr-15	*Citrus sinensis*	I	V	Quillota	-71,194653	-32,872836	165	C	HCOI60	H28S43	19633	INRA	-	KY085165	KY085772
Saissetia oleae	C	Apr-15	*Citrus sinensis*	I	V	Quillota	-71,195847	-32,873494	158	C	HCOI61	H28S38	19657	INRA	-	KY085188	KY085714
Saissetia oleae	C	Apr-15	*Pouteria lucuma*	N2	V	Quillota	-71,191833	-32,875178	170	C	HCOI61	H28S38	19646	INRA	-	KY085178	KY085703
Saissetia oleae	C	Apr-15	*Pouteria lucuma*	N2	V	Quillota	-71,191833	-32,875178	170	C	HCOI61	H28S39	19647	INRA	-	KY085179	KY085731
Saissetia oleae	C	Apr-15	*Prunus persica*	I	V	Quillota	-71,191958	-32,875394	170	C	HCOI61	H28S39	19644	INRA	-	KY085176	KY085737
Saissetia oleae	C	Apr-15	*Prunus persica*	I	V	Quillota	-71,191958	-32,875394	170	C	HCOI63	-	19645	INRA	-	KY085177	-
Saissetia oleae	C	Jul-15	*Abutilon* sp.	I	V	Concon	-71,538108	-32,934253	65	U	-	H28S38	24161	INRA	-	-	KY085716
Saissetia oleae	C	Jul-15	*Abutilon* sp.	I	V	Concon	-71,538108	-32,934253	65	U	HCOI61	H28S39	24162	INRA	-	KY085253	KY085746
Saissetia oleae	C	Sep-15	*Nerium oleander*	I	Met	Til Til	-70,927575	-33,114133	564	U	-	H28S38	24218	INRA	-	-	KY085725
Saissetia oleae	C	Sep-15	*Nerium oleander*	I	Met	Til Til	-70,927575	-33,114133	564	U	-	H28S43	24217	INRA	-	-	KY085783
Saissetia oleae	C	Sep-15	*Olea europaea*	I	V	Alborrogo	-71,670617	-33,365639	40	U	-	H28S38	24209	INRA	-	-	KY085730
Saissetia oleae	C	Sep-15	*Tristerix tetrandrus*	N2	V	Alborrogo	-71,670617	-33,365639	40	U	-	H28S38	24212	INRA	-	-	KY085717
Saissetia oleae	C	Sep-15	*Olea europaea*	I	V	Alborrogo	-71,670617	-33,365639	40	U	-	H28S41	24210	INRA	-	-	KY085754
Saissetia oleae	C	Sep-15	*Tristerix tetrandrus*	N2	V	Alborrogo	-71,670617	-33,365639	40	U	-	H28S41	24211	INRA	-	-	KY085756
Saissetia oleae	C	Mar-15	*Olea europaea*	I	Met	Providencia	-70,632794	-33,426369	810	U	HCOI61	H28S38	19687	INRA	-	KY085216	KY085689
Saissetia oleae	C	Mar-15	*Olea europaea*	I	Met	Providencia	-70,632794	-33,426369	810	U	HCOI61	H28S38	19688	INRA	-	KY085217	KY085697
Saissetia oleae	C	Mar-15	*Olea europaea*	I	Met	Santiago	-70,643983	-33,441006	595	U	HCOI61	H28S38	19696	INRA	-	KY085225	KY085711
Saissetia oleae	C	Mar-15	*Olea europaea*	I	Met	Santiago	-70,643983	-33,441006	595	U	HCOI61	H28S39	19695	INRA	-	KY085224	KY085741
Saissetia oleae	C	May-15	*Schinus latifolius*	N2	V	Casablanca	-71,394678	-33,457094	241	C	HCOI61	H28S39	19636	INRA	-	KY085168	KY085738
Saissetia oleae	C	May-15	*Schinus latifolius*	N2	V	Casablanca	-71,394678	-33,457094	241	C	HCOI61	H28S39	19637	INRA	-	KY085169	KY085740
Saissetia oleae	C	May-15	*Schinus latifolius*	N2	V	Cartagena	-71,419708	-33,482900	218	C	HCOI61	H28S38	19611	INRA	-	KY085145	KY085698
Saissetia oleae	C	May-15	*Schinus latifolius*	N2	V	Cartagena	-71,419708	-33,482900	218	C	HCOI61	H28S43	19612	INRA	-	KY085146	KY085759
Saissetia oleae	C	May-15	*Olea europaea*	I	Met	La Pintana	-70,634269	-33,568869	623	U	HCOI61	H28S38	19640	INRA	-	KY085172	KY085712
Saissetia oleae	C	May-15	*Olea europaea*	I	Met	La Pintana	-70,634269	-33,568869	623	U	HCOI61	H28S38	19641	INRA	-	KY085173	KY085707
Saissetia oleae	C	May-15	*Pyrus communis*	I	Met	Santiago	-70,633558	-33,569242	625	U	HCOI61	H28S38	19649	INRA	-	KY085181	KY085688
Saissetia oleae	C	May-15	*Pyrus communis*	I	Met	Santiago	-70,633558	-33,569242	625	U	HCOI61	-	19648	INRA	-	KY085180	-

Species name	Family	Sample Date	Host plant	Type	Region	Municipality	Longitude	Latitude	Altitude (m.)	Area	Haplotype COI	Haplotype 28S	Molecular Code	Voucher Location	Voucher Code	GenBank access. COI	GenBank access. 28S
Saissetia oleae	C	Mar-15	Unknow	-	Met	Nueva Independencia	-70,762842	-33,643031	510	U	HCOI61	H28S37	19704	INRA	-	KY085233	KY085682
Saissetia oleae	C	Mar-15	Unknow	-	Met	Nueva Independencia	-70,762842	-33,643031	510	U	HCOI61	-	19703	INRA	-	KY085232	-
Saissetia oleae	C	Mar-15	*Nerium oleander*	I	Met	San Bernando	-70,722472	-33,669439	544	U	HCOI61	H28S38	19673	INRA	-	KY085202	KY085713
Saissetia oleae	C	Mar-15	*Aesculus hippocastanum*	I	Met	San Bernando	-70,722472	-33,669439	544	U	HCOI60	H28S43	19683	INRA	-	KY085212	KY085768
Saissetia oleae	C	Mar-15	*Aesculus hippocastanum*	I	Met	San Bernando	-70,722472	-33,669439	544	U	HCOI60	-	19684	INRA	-	KY085213	-
Saissetia oleae	C	Dec-15	*Maytenus boaria*	N2	Met	San José de Maipo	-70,340842	-33,678653	1005	N	-	H28S43	24312	INRA	-	-	KY085802
Saissetia oleae	C	Dec-15	*Maytenus boaria*	N2	Met	San José de Maipo	-70,340842	-33,678653	1005	N	-	H28S43	24311	INRA	-	-	KY085798
Saissetia oleae	C	May-15	*Prunus domestica*	I	Met	Paine	-70,803033	-33,831142	387	C	HCOI61	H28S38	19593	INRA	-	KY085129	KY085699
Saissetia oleae	C	May-15	*Prunus domestica*	I	Met	Paine	-70,803033	-33,831142	387	C	HCOI61	H28S39	19592	INRA	-	KY085128	KY085739
Saissetia oleae	C	May-15	*Cydonia oblonga*	I	Met	Paine	-70,796336	-33,833192	372	C	HCOI61	H28S38	19609	INRA	-	KY085143	KY085694
Saissetia oleae	C	May-15	*Cydonia oblonga*	I	Met	Paine	-70,796336	-33,833192	372	C	HCOI61	H28S43	19610	INRA	-	KY085144	KY085776
Saissetia oleae	C	Jan-16	*Olea europaea*	I	Met	Paine	-70,764017	-33,851461	380	U	-	H28S38	24327	INRA	-	-	KY085723
Saissetia oleae	C	Jan-16	*Olea europaea*	I	Met	Paine	-70,764017	-33,851461	380	U	-	H28S39	24328	INRA	-	-	KY085743
Saissetia oleae	C	Jan-16	*Citrus limon*	I	Met	Paine	-70,763742	-33,854836	375	U	-	H28S39	24325	INRA	-	-	KY085748
Saissetia oleae	C	Jan-16	*Citrus limon*	I	Met	Paine	-70,763742	-33,854836	375	U	-	H28S39	24326	INRA	-	-	KY085751
Saissetia oleae	C	Oct-15	*Citrus sinensis*	I	VI	Coya	-70,530786	-34,206528	786	U	HCOI63	H28S43	24253	INRA	-	KY085302	KY085799
Saissetia oleae	C	Oct-15	*Nerium oleander*	I	VI	Rengo	-70,845967	-34,356319	338	U	-	H28S37	24799	INRA	-	-	KY085675
Saissetia oleae	C	Oct-15	*Nerium oleander*	I	VI	Rengo	-70,845967	-34,356319	338	U	HCOI60	H28S38	24284	INRA	-	KY085325	KY085718
Saissetia oleae	C	Oct-15	*Nerium oleander*	I	VI	Rengo	-70,845967	-34,356319	338	U	-	H28S38	24800	INRA	-	-	KY085686
Saissetia oleae	C	Oct-15	*Nerium oleander*	I	VI	Rengo	-70,845967	-34,356319	338	U	-	H28S40	24285	INRA	-	-	KY085752
Saissetia oleae	C	Oct-15	*Nerium oleander*	I	VI	Rengo	-70,845967	-34,356319	338	U	-	H28S42	27882	INRA	-	-	KY085758
Saissetia oleae	C	Oct-15	*Nerium oleander*	I	VI	Rengo	-70,845967	-34,356319	338	U	-	H28S43	27883	INRA	-	-	KY085760
Saissetia oleae	C	Mar-15	*Citrus sp.*	I	VI	Pelequen	-70,923022	-34,434842	278	C	HCOI61	H28S36	19700	INRA	-	KY085229	KY085672
Saissetia oleae	C	Mar-15	*Citrus sp.*	I	VI	Pelequen	-70,923022	-34,434842	278	C	HCOI61	H28S37	19699	INRA	-	KY085228	KY085678
Saissetia oleae	C	Mar-15	Unknow	-	VI	Pelequen	-70,923022	-34,434842	278	C	HCOI61	H28S38	19697	INRA	-	KY085226	KY085695
Saissetia oleae	C	Mar-15	Unknow	-	VI	Pelequen	-70,923022	-34,434842	278	C	HCOI61	H28S38	19698	INRA	-	KY085227	KY085705
Saissetia oleae	C	Mar-15	*Schinus latifolius*	N2	VI	San Fernando	-70,922308	-34,507467	313	U	HCOI60	H28S36	19671	INRA	-	KY085200	KY085671
Saissetia oleae	C	Mar-15	*Schinus latifolius*	N2	VI	San Fernando	-70,922308	-34,507467	313	U	HCOI60	-	19672	INRA	-	KY085201	-
Saissetia oleae	C	Mar-15	*Citrus sinensis*	I	VI	Placilla	-71,124206	-34,625253	254	C	HCOI63	H28S37	19678	INRA	-	KY085207	KY085681
Saissetia oleae	C	Mar-15	*Citrus sinensis*	I	VI	Placilla	-71,124206	-34,625253	254	C	HCOI63	H28S37	19677	INRA	-	KY085206	KY085680
Saissetia oleae	C	Oct-15	*Citrus sinensis*	I	VI	Santa Cruz	-71,366272	-34,638958	175	U	HCOI63	H28S39	24257	INRA	-	KY085305	KY085744
Saissetia oleae	C	Oct-15	*Citrus sinensis*	I	VI	Santa Cruz	-71,366272	-34,638958	175	U	-	H28S39	24258	INRA	-	-	KY085749
Saissetia oleae	C	Feb-15	*Pyracantha sp.*	I	VI	Chimbarongo	-70,988672	-34,642783	376	U	HCOI63	H28S39	19708	INRA	-	KY085236	KY085732
Saissetia oleae	C	Feb-15	*Pyracantha sp.*	I	VI	Chimbarongo	-70,988672	-34,642783	376	U	HCOI58	-	19709	INRA	-	KY085237	-
Saissetia oleae	C	Jan-15	*Citrus aurantium*	I	VI	Nancagua	-71,203981	-34,651092	220	U	HCOI63	H28S37	19710	INRA	-	KY085238	KY085677
Saissetia oleae	C	Jan-16	*Olea europaea*	I	VI	Santa Cruz	-71,343717	-34,653167	177	U	-	H28S39	24316	INRA	-	-	KY085747
Saissetia oleae	C	Feb-15	*Olea europaea*	I	V	Quillota	-71,251956	-34,901750	235	U	HCOI60	H28S38	19606	INRA	-	KY085140	KY085691
Saissetia oleae	C	May-15	*Nerium oleander*	I	VII	Curico	-71,206742	-34,978044	234	U	HCOI61	H28S43	19580	INRA	-	KY085116	KY085763
Saissetia oleae	C	May-15	*Nerium oleander*	I	VII	Curico	-71,206742	-34,978044	234	U	HCOI61	-	19581	INRA	-	KY085117	-
Saissetia oleae	C	Oct-15	*Citrus sinensis*	I	VII	Curico	-71,221636	-34,983642	223	U	-	H28S39	24246	INRA	-	-	KY085745
Saissetia oleae	C	Oct-15	*Citrus sinensis*	I	VII	Curico	-71,221636	-34,983642	223	U	-	H28S43	24247	INRA	-	-	KY085792
Saissetia oleae	C	Mar-15	*Robinia pseudoacacia*	I	VII	Sagrada Familia	-71,306525	-35,034678	200	C	HCOI61	H28S39	19681	INRA	-	KY085210	KY085742
Saissetia oleae	C	Mar-15	*Robinia pseudoacacia*	I	VII	Sagrada Familia	-71,306525	-35,034678	200	C	HCOI63	-	19682	INRA	-	KY085211	-
Saissetia oleae	C	Mar-15	*Olea europaea*	I	VII	Sagrada Familia	-71,624278	-35,132542	96	C	HCOI61	H28S38	19686	INRA	-	KY085215	KY085715
Saissetia oleae	C	Mar-15	*Olea europaea*	I	VII	Sagrada Familia	-71,624278	-35,132542	96	C	HCOI61	H28S43	19685	INRA	-	KY085214	KY085778
Saissetia oleae	C	May-15	*Fraxinus sp.*	I	VII	Linares	-71,616908	-35,842264	156	U	HCOI61	H28S38	19576	INRA	-	KY085112	KY085709
Saissetia oleae	C	May-15	*Nerium oleander*	I	VII	Linares	-71,616908	-35,842264	156	U	HCOI60	H28S38	19607	INRA	-	KY085141	KY085704
Saissetia oleae	C	May-15	*Quillaja saponaria*	N1	VII	Linares	-71,616908	-35,842264	156	U	HCOI61	H28S38	19604	INRA	-	KY085139	KY085692

Species name	Family	Sample Date	Host plant	Type	Region	Municipality	Longitude	Latitude	Altitude (m.)	Area	Haplotype COI	Haplotype 28S	Molecular Code	Voucher Location	Voucher Code	GenBank access. COI	GenBank access. 28S
Saissetia oleae	C	May-15	*Quillaja saponaria*	N1	VII	Linares	-71,616908	-35,842264	156	U	HCOI61	H28S39	19603	INRA	-	KY085138	KY085733
Saissetia oleae	C	May-15	*Fraxinus sp.*	I	VII	Linares	-71,616908	-35,842264	156	U	HCOI60	H28S43	19577	INRA	-	KY085113	KY085775
Saissetia oleae	C	May-15	*Nerium oleander*	I	VII	Linares	-71,616908	-35,842264	156	U	HCOI60	-	19608	INRA	-	KY085142	-
Saissetia oleae	C	May-15	*Citrus limon*	I	VII	Parral	-71,819258	-36,147072	179	U	HCOI61	H28S38	19586	INRA	-	KY085122	KY085708
Saissetia oleae	C	May-15	*Citrus limon*	I	VII	Parral	-71,819258	-36,147072	179	U	HCOI63	H28S43	19587	MNHN	1600307	KY085123	KY085777
Saissetia oleae	C	May-15	*Nerium oleander*	I	VII	Parral	-71,821250	-36,149314	182	U	HCOI61	H28S43	19575	INRA	-	KY085111	KY085770
Saissetia oleae	C	May-15	*Nerium oleander*	I	VII	Parral	-71,821250	-36,149314	182	U	HCOI61	H28S43	19574	INRA	-	KY085110	KY085773
Saissetia oleae	C	May-15	*Maytenus boaria*	N2	VIII	San Carlos	-71,961911	-36,432956	181	U	HCOI60	-	19617	INRA	-	KY085149	-
Saissetia oleae	C	May-15	*Maytenus boaria*	N2	VIII	San Carlos	-71,961911	-36,432956	181	U	HCOI60	-	19618	INRA	-	KY085150	-
Saissetia oleae	C	May-15	*Olea europaea*	I	VIII	San Carlos	-71,962781	-36,437169	179	U	HCOI61	H28S39	19579	INRA	-	KY085115	KY085736
Saissetia oleae	C	May-15	*Olea europaea*	I	VIII	San Carlos	-71,962781	-36,437169	179	U	HCOI61	H28S43	19578	INRA	-	KY085114	KY085769
Saissetia oleae	C	May-15	*Choisya ternata*	I	VIII	Chillan	-72,071822	-36,593292	148	U	HCOI59	H28S37	19591	MNHN	1600306	KY085127	KY085683
Saissetia oleae	C	May-15	*Choisya ternata*	I	VIII	Chillan	-72,071822	-36,593292	148	U	HCOI59	H28S40	19590	INRA	-	KY085126	KY085753
Saissetia oleae	C	May-15	*Laurus nobilis*	I	VIII	Chillan	-72,099961	-36,595719	129	U	HCOI60	H28S37	19660	INRA	-	KY085191	KY085684
Saissetia oleae	C	May-15	*Laurus nobilis*	I	VIII	Chillan	-72,099961	-36,595719	129	U	HCOI61	H28S38	19661	INRA	-	KY085192	KY085696

Present Study - 28S				GenBank database (Best hit)						
Family	Morphological identification	Molecular Code	Haplotype 28S	sscinames	length	sseqid	evalue	bitscore	qcovs	pident
D	*Aonidiella aurantii*	19643	H28S24	*Aonidiella environmental sample*	700	KF887372.1	0.0	1293	97	100.00
D	*Aonidiella ensifera*	24333	H28S13	*Diaspidiotus sp. GEM-2005c*	602	GQ325476.1	0.0	1077	95	99.00
D	*Aonidiella ensifera*	19391	H28S14	*Diaspidiotus sp. GEM-2005c*	703	GQ325476.1	0.0	1264	95	99.15
D	*Aonidomytilus sp.*	24303	H28S07	*Dactylaspis sp. GEM-2005b*	676	DQ145312.2	0.0	1249	95	100.00
D	*Aspidiotus nerii*	19392	H28S33	*Aspidiotus nerii*	736	GU213889.1	0.0	1360	100	100.00
D	*Aspidiotus nerii*	19474	H28S34	*Aspidiotus nerii*	711	GU213889.1	0.0	1314	100	100.00
D	*Aspidiotus nerii*	19508	H28S32	*Aspidiotus nerii*	736	GU213889.1	0.0	1354	100	99.86
D	*Chrysomphalus dictyospermi*	24320	H28S23	*Chrysomphalus dictyospermi*	701	GQ325463.1	0.0	1277	95	99.57
D	*Diaspidiotus ancylus*	19538	H28S27	*Abgrallaspis perseae*	734	FJ040865.1	0.0	1306	100	98.77
D	*Diaspidiotus perniciosus*	19564	H28S26	*Abgrallaspis perseae*	735	FJ040865.1	0.0	1267	100	97.82
D	*Diaspidiotus perniciosus*	24353	H28S25	*Abgrallaspis perseae*	735	FJ040865.1	0.0	1273	100	97.96
D	*Diaspis chilensis*	19501	H28S09	*Diaspis miranda*	722	FJ040867.1	0.0	1221	100	97.23
D	*Diaspis echinocacti*	19389	H28S11	*Diaspis echinocacti;Pulvinaria psidii*	720	JQ651182.1	0.0	1303	100	99.31
D	*Epidiaspis leperii*	19386	H28S10	*Epidiaspis leperii*	688	DQ145324.2	0.0	1249	95	99.27
D	*Furchadaspis zamiae*	19407	H28S08	*Furchadaspis zamiae*	689	DQ145333.2	0.0	1254	95	99.56
D	*Hemiberlesia lataniae*	24331	H28S28	*Hemiberlesia lataniae*	677	FJ040869.1	0.0	1251	100	100.00
D	*Hemiberlesia lataniae*	19467	H28S29	*Hemiberlesia lataniae*	733	FJ040869.1	0.0	1354	100	100.00
D	*Hemiberlesia lataniae*	19416	H28S30	*Hemiberlesia lataniae*	733	FJ040869.1	0.0	1347	100	99.86
D	*Hemiberlesia lataniae*	19453	H28S31	*Hemiberlesia nr. Lataniae*	733	FJ040870.1	0.0	1349	100	99.86
D	*Hemiberlesia palmae*	24286	H28S22	*Abgrallaspis cyanophylli*	657	JQ651299.1	0.0	1192	97	99.39
D	*Hemiberlesia rapax*	19442	H28S20	*Hemiberlesia rapax*	703	DQ145343.2	0.0	1275	95	99.43
D	*Hemiberlesia rapax*	24381	H28S15	*Hemiberlesia rapax*	647	DQ145343.2	0.0	1179	95	99.54
D	*Hemiberlesia rapax*	19492	H28S17	*Hemiberlesia rapax*	703	DQ145343.2	0.0	1282	95	99.57
D	*Hemiberlesia rapax*	19521	H28S18	*Hemiberlesia rapax*	703	DQ145343.2	0.0	1282	95	99.57
D	*Hemiberlesia rapax*	19432	H28S21	*Hemiberlesia rapax*	703	DQ145343.2	0.0	1282	95	99.57
D	*Hemiberlesia rapax*	24355	H28S16	*Hemiberlesia rapax*	647	DQ145343.2	0.0	1184	95	99.69
D	*Hemiberlesia rapax*	19547	H28S19	*Hemiberlesia rapax*	703	DQ145343.2	0.0	1288	95	99.72
D	*Lepidosaphes beckii*	19437	H28S01	*Lepidosaphes beckii*	682	DQ145352.2	0.0	1260	95	100.00
D	*Lepidosaphes beckii*	19510	H28S02	*Lepidosaphes beckii*	682	DQ145352.2	0.0	1242	95	99.56
D	*Lepidosaphes ulmi*	19475	H28S04	*Lepidosaphes beckii*	686	DQ145352.2	0.0	1051	95	94.46
D	*Lepidosaphes ulmi*	19531	H28S03	*Lepidosaphes beckii*	684	DQ145352.2	0.0	1066	95	94.88
D	*Melanaspis sitreana*	19613	H28S12	*Melanaspis environmental sample*	728	KF887365.1	0.0	1234	100	97.25
D	*Pseudoparlatoria chilina*	24294	H28S05	*Pinnaspis strachani*	720	FJ040871.1	0.0	1042	99	93.06
D	*Pseudoparlatoria sp.*	24290	H28S06	*Prodigiaspis sp. GEM-2005*	691	DQ145379.2	0.0	1134	95	96.38

Present Study - 28S				GenBank database (Best hit)						
Family	Morphological identification	Molecular Code	Haplotype 28S	sscinames	length	sseqid	evalue	bitscore	qcovs	pident
C	*Ceroplastes sinensis*	19662	H28S49	*Ceroplastes ceriferus*	826	JF719820.1	0.0	1223	100	93.83
C	*Ceroplastes sp.I*	19600	H28S48	*Ceroplastes ceriferus*	826	JF719820.1	0.0	1229	100	93.95
C	*Ceroplastes sp.I*	19669	H28S47	*Ceroplastes ceriferus*	826	JF719820.1	0.0	1234	100	94.07
C	*Coccus hesperidum*	19615	H28S51	*Coccus formicarii*	805	JX866687.1	0.0	1447	100	99.13
C	*Parasaissetia nigra*	19721	H28S50	*Parasaissetia nigra*	735	JQ651215.1	0.0	1358	91	100.00
C	*Parthenolecanium corni*	19596	H28S52	*Parthenolecanium corni*	704	KP189587.1	0.0	1301	86	100.00
C	*Protopulvinaria pyriformis*	24178	H28S53	*Protopulvinaria pyriformis*	740	JX866698.1	0.0	1354	95	99.73
C	*Protopulvinaria pyriformis*	19431	H28S54	*Protopulvinaria pyriformis*	758	JX866698.1	0.0	1387	96	99.74
C	*Pulvinariella mesembryanthemi*	24300	H28S55	*Milviscutulus mangiferae*	779	JX645355.1	0.0	861	96	87.29
C	*Saissetia coffeae*	19619	H28S46	*Saissetia coffeae*	798	JX645353.1	0.0	1458	99	99.62
C	*Saissetia coffeae*	24305	H28S45	*Saissetia coffeae*	798	JX645353.1	0.0	1469	99	99.87
C	*Saissetia oleae*	24229	H28S42	*Saissetia miranda*	767	JX866694.1	0.0	1362	96	98.70
C	*Saissetia oleae*	19612	H28S43	*Saissetia miranda*	767	JX866694.1	0.0	1367	96	98.83
C	*Saissetia oleae*	24285	H28S40	*Saissetia miranda*	767	JX866694.1	0.0	1373	96	98.96
C	*Saissetia oleae*	24210	H28S41	*Saissetia miranda*	767	JX866694.1	0.0	1373	96	98.96
C	*Saissetia oleae*	19622	H28S44	*Saissetia miranda*	767	JX866694.1	0.0	1373	96	98.96
C	*Saissetia oleae*	19573	H28S38	*Saissetia miranda*	767	JX866694.1	0.0	1378	96	99.09
C	*Saissetia oleae*	19717	H28S36	*Saissetia miranda*	767	JX866694.1	0.0	1384	96	99.22
C	*Saissetia oleae*	19647	H28S39	*Saissetia miranda*	767	JX866694.1	0.0	1384	96	99.22
C	*Saissetia oleae*	19667	H28S37	*Saissetia miranda*	767	JX866694.1	0.0	1389	96	99.35

Present Study - COI				GenBank database (Best hit)						
Family	Morphological identification	Molecular Code	Haplotype 28S	sscinames	length	sseqid	evalue	bitscore	qcovs	pident
D	*Aonidiella aurantii*	19514	HCOI40	Aonidiella aurantii	649	HM474068.1	0.0	1199	100	100.00
D	*Aonidiella aurantii*	19642	HCOI41	Aonidiella aurantii	649	HM474068.1	0.0	1188	100	99.69
D	*Aonidiella ensifera*	24334	HCOI54	Aonidiella aurantii	644	HM474068.1	0.0	869	99	90.99
D	*Aonidomytilus sp.*	24303	HCOI63	Kuwanaspis hikosani	653	HM474202.1	0.0	734	100	87.14
D	*Aonidomytilus sp.*	24301	HCOI64	Kuwanaspis hikosani	653	HM474202.1	0.0	734	100	87.14
D	*Aspidiotus nerii* I	19516	HCOI32	Aspidiotus nerii	644	HM474081.1	0.0	941	99	93.01
D	*Aspidiotus nerii* I	24168	HCOI33	Aspidiotus nerii	644	HM474081.1	0.0	935	99	92.86
D	*Aspidiotus nerii* II	19393	HCOI34	Aspidiotus nerii	327	HM474081.1	7E-80	309	36	84.10
D	*Aspidiotus nerii* II	24242	HCOI35	Aspidiotus nerii	325	HM474081.1	7E-80	309	36	84.00
D	*Aspidiotus nerii* II	24232	HCOI36	Aspidiotus nerii	325	HM474081.1	7E-80	309	36	84.00
D	*Aspidiotus nerii* II	19462	HCOI37	Aspidiotus nerii	322	HM474081.1	3E-78	303	36	83.85
D	*Aspidiotus nerii* III	19565	HCOI27	Aspidiotus nerii	649	HM474081.1	0.0	1199	100	100.00
D	*Aspidiotus nerii* III	24369	HCOI28	Aspidiotus nerii	649	HM474081.1	0.0	1194	100	99.85
D	*Aspidiotus nerii* III	24338	HCOI29	Aspidiotus nerii	649	HM474081.1	0.0	1194	100	99.85
D	*Aspidiotus nerii* III	24363	HCOI30	Aspidiotus nerii	649	HM474081.1	0.0	1149	100	98.61
D	*Aspidiotus nerii* III	24344	HCOI31	Aspidiotus nerii	649	HM474081.1	0.0	1144	100	98.46
D	*Chrysomphalus dictyospermi*	19488	HCOI44	Chrysomphalus dictyospermi	649	JQ267366.1	0.0	1188	100	99.69
D	*Diaspidiotus ancylus*	19538	HCOI55	Chionaspis pinifoliae	651	KR044519.1	0.0	900	100	91.71
D	*Diaspidiotus ancylus*	19555	HCOI56	Chionaspis pinifoliae	651	KR044519.1	0.0	889	100	91.40
D	*Diaspidiotus perniciosus*	19505	HCOI43	Diaspidiotus perniciosus	649	HM474184.1	0.0	1182	100	99.54
D	*Diaspis chilensis*	19501	HCOI66	Chionaspis pinifoliae	649	KR038420.1	0.0	806	99	89.21
D	*Diaspis chilensis*	19705	HCOI67	Aulacaspis rosarum	647	HM474087.1	0.0	802	99	89.18
D	*Diaspis chilensis*	19427	HCOI68	Aulacaspis rosarum	647	HM474087.1	0.0	802	99	89.18
D	*Diaspis chilensis*	19426	HCOI69	Aulacaspis spinosa	646	HM474088.1	0.0	802	99	89.16
D	*Diaspis echinocacti*	19389	HCOI70	Chionaspis pinifoliae	652	KR038629.1	0.0	750	100	87.42
D	*Epidiaspis leperii*	19405	HCOI65	Chionaspis pinifoliae	650	KR041932.1	0.0	706	100	86.31
D	*Furchadaspis zamiae*	19406	HCOI25	Chionaspis pinifoliae	446	KR042165.1	2E-139	507	100	87.22
D	*Furchadaspis zamiae*	19407	HCOI26	Chionaspis pinifoliae	446	KR042165.1	2E-139	507	100	87.22
D	*Hemiberlesia lataniae*	19527	HCOI49	Hemiberlesia lataniae	647	HQ179913.1	0.0	869	99	90.88
D	*Hemiberlesia lataniae*	19536	HCOI50	Hemiberlesia lataniae	647	HQ179913.1	0.0	874	99	91.04
D	*Hemiberlesia lataniae*	19512	HCOI51	Hemiberlesia lataniae	647	HQ179913.1	0.0	874	99	91.04
D	*Hemiberlesia palmae*	24286	HCOI42	Aonidiella aurantii	644	HM474068.1	0.0	880	99	91.30
D	*Hemiberlesia rapax* I	19432	HCOI45	Aonidiella aurantii	649	HM474068.1	0.0	889	100	91.37
D	*Hemiberlesia rapax* II	24356	HCOI46	*Aonidiella citrina*	649	JQ267365.1	0.0	889	100	91.37
D	*Hemiberlesia rapax* II	27906	HCOI47	*Aonidiella citrina*	649	JQ267365.1	0.0	883	100	91.22
D	*Hemiberlesia rapax* II	27914	HCOI48	*Aonidiella citrina*	649	JQ267365.1	0.0	900	100	91.68
D	*Hemiberlesia rapax* III	19440	HCOI52	*Aspidiotus excisus*	559	HM474079.1	3E-152	549	83	84.97
D	*Hemiberlesia rapax* III	19547	HCOI53	*Aspidiotus excisus*	560	HM474079.1	1E-150	544	83	84.82

Present Study - COI				GenBank database (Best hit)						
Family	Morphological identification	Molecular Code	Haplotype 28S	sscinames	length	sseqid	evalue	bitscore	qcovs	pident
D	*Lepidosaphes beckii*	19543	HCOI59	*Lepidosaphes beckii*	649	JQ267367.1	0.0	1188	100	99.69
D	*Lepidosaphes beckii*	19388	HCOI60	*Lepidosaphes beckii*	649	JQ267367.1	0.0	1182	100	99.54
D	*Lepidosaphes beckii*	24271	HCOI61	*Lepidosaphes beckii*	649	JQ267367.1	0.0	1199	100	100.00
D	*Lepidosaphes beckii*	19437	HCOI62	*Lepidosaphes beckii*	649	JQ267367.1	0.0	1194	100	99.85
D	*Lepidosaphes ulmi*	19530	HCOI57	*Lepidosaphes kamakurensis*	648	HM474209.1	0.0	771	99	88.12
D	*Lepidosaphes ulmi*	19557	HCOI58	*Lepidosaphes kamakurensis*	648	HM474209.1	0.0	765	99	87.96
D	*Melanaspis sitreana*	19522	HCOI38	*Aspidiotus excisus*	647	HM474079.1	0.0	791	99	88.87
D	*Melanaspis sitreana*	19523	HCOI39	*Aspidiotus excisus*	647	HM474079.1	0.0	785	99	88.72
D	*Pseudoparlatoria chilina*	24294	HCOI71	*Aspidiotus excisus*	650	HM474077.1	0.0	667	99	85.54
C	*Ceroplastes sinensis*	19662	HCOI07	Ceroplastes rubens	648	KP981081.1	0.0	782	99	88.43
C	*Ceroplastes* sp.I	19669	HCOI08	Ceroplastes sp. DSP-2010	648	GU936955.1	0.0	754	99	87.65
C	*Ceroplastes* sp.II	24170	HCOI09	Ceroplastes rubens	649	KP981081.1	0.0	706	100	86.29
C	*Coccidae unidentified*	24335	HCOI24	Eulecanium cerasorum	541	KJ908688.1	3E-127	466	82	82.44
C	*Coccus hesperidum*	19723	HCOI15	Unaspis yanonensis	649	KP981079.1	0.0	1194	100	99.85
C	*Coccus hesperidum*	24292	HCOI16	Unaspis yanonensis	649	KP981079.1	0.0	1199	100	100.00
C	*Coccus hesperidum*	24163	HCOI17	Coccus hesperidum	648	GU936950.1	0.0	1197	99	100.00
C	*Coccus hesperidum*	19615	HCOI18	Unaspis yanonensis	649	KP981079.1	0.0	1149	100	98.61
C	*Coccus hesperidum*	19707	HCOI19	Unaspis yanonensis	649	KP981079.1	0.0	1138	100	98.31
C	*Coccus hesperidum*	24339	HCOI20	Unaspis yanonensis	649	KP981079.1	0.0	1144	100	98.46
C	*Parasaissetia nigra*	19721	HCOI14	*Parasaissetia nigra*	543	KP189867.1	0.0	1003	83	100.00
C	*Parthenolecanium corni*	24245	HCOI21	*Parthenolecanium corni*	543	JQ795616.1	0.0	998	83	99.82
C	*Parthenolecanium corni*	19596	HCOI22	*Parthenolecanium corni*	543	JQ795616.1	0.0	992	83	99.63
C	*Parthenolecanium corni*	24215	HCOI23	*Parthenolecanium corni*	543	KP189848.1	0.0	1003	83	100.00
C	*Protopulvinaria pyriformis*	19431	HCOI11	*Protopulvinaria pyriformis*	579	JX853912.1	0.0	1070	89	100.00
C	*Protopulvinaria pyriformis*	24342	HCOI12	*Protopulvinaria pyriformis*	579	JX853912.1	0.0	1070	89	100.00
C	*Pulvinariella mesembryanthemi*	24300	HCOI13	*Ceroplastes rusci*	643	KJ919960.1	0.0	682	98	85.85
C	*Saissetia coffeae*	19619	HCOI10	*Ceroplastes sp. DSP-2010*	647	GU936955.1	0.0	730	99	87.02
C	*Saissetia oleae*	19645	HCOI01	*Saissetia sp. DSP-2010*	647	GU936954.1	0.0	935	99	92.74
C	*Saissetia oleae*	19659	HCOI02	*Saissetia sp. DSP-2010*	647	GU936954.1	0.0	941	99	92.89
C	*Saissetia oleae*	19715	HCOI03	*Saissetia sp. DSP-2010*	647	GU936954.1	0.0	935	99	92.74
C	*Saissetia oleae*	19676	HCOI04	*Saissetia sp. DSP-2010*	647	GU936954.1	0.0	924	99	92.43
C	*Saissetia oleae*	19590	HCOI05	*Saissetia sp. DSP-2010*	647	GU936954.1	0.0	935	99	92.74
C	*Saissetia oleae*	19709	HCOI06	*Saissetia sp. DSP-2010*	647	GU936954.1	0.0	924	99	92.43

Genus	*Annona*	*Laureliopsis*	*Cryptocarya Laurus Persea*	*Peumus*	*Yucca*	*Berberis*	*Macadamia*	*Vitis*	*Aextoxicon*	*Ilex*	*Euonymus Maytenus*	*Robinia*	*Acacia Prosopis*	*Quillaja*	*Ficus*	*Cydonia Pyrus Malus Prunus Pyracantha*	*Nothofagus*	*Juglans*	*Punica*	-	*Schinus*	*Choisya Citrus*	*Aesculus*	*Aristotelia*	*Abutilon*	*Tristerix*	*Echinopsis*	*Vaccinium*	*Pouteria*	*Nerium*	*Fraxinus Oleae*	*Hedera Schefflera*			
**Order**	**Magnoliales**	**Laurales**			**Asparagales**	**Ranunculales**	**Proteales**	**Vitales**	**Celastrales**			**Fabales**			**Rosales**		**Fagales**		**Myrtales**		**Sapindales**			**Malvales**		**Santalales**	**Caryophyllales**	**Ericales**		**Gentianales**	**Lamiales**	**Apiales**			
**Family**	**Annonaceae**	**Atherospermataceae**	**Lauraceae**	**Monimiaceae**	**Asparagaceae**	**Berberidaceae**	**Proteaceae**	**Vitaceae**	**Aextoxicaceae**	**Aquifoliaceae**	**Celastraceae**	**Fabaceae**	**Mimosaceae**	**Quillajaceae**	**Moraceae**	**Rosaceae**	**Nothofagaceae**	**Juglandaceae**	**Lythraceae**	**Myrtaceae**	**Anacardiaceae**	**Rutaceae**	**Sapindaceae**	**Elaeocarpaceae**	**Malvaceae**	**Loranthaceae**	**Cactaceae**	**Ericaceae**	**Sapotaceae**	**Apocynaceae**	**Oleaceae**	**Araliaceae**	**Nb Species**	**Nb Family**	**Nb Orden**
Aonidiella aurantii	-	-	-	-	-	-	-	-	-	-	-	-	-	-	-	-	-	-	-	-	-	2	-	-	-	-	-	-	-	-	-	-	**2**	**1**	**1**
Aonidiella ensifera	-	-	-	-	-	-	-	-	-	-	-	-	-	-	-	-	-	-	-	-	-	-	-	-	-	-	-	1	-	-	1	1	**3**	**3**	**3**
Aonidomytilus sp.	-	-	-	-	-	-	-	-	-	1	-	-	-	-	-	-	-	-	-	-	-	-	-	-	-	-	-	-	-	-	-	-	**1**	**1**	**1**
Aspidiotus nerii I	-	-	2	-	-	-	-	-	-	1	-	-	1	-	-	-	-	-	-	-	-	-	-	1	-	-	-	-	-	-	1	1	**7**	**6**	**6**
Aspidiotus nerii II	-	-	1	-	-	-	-	-	-	-	1	-	-	-	-	-	-	-	-	-	1	-	1	-	-	-	-	-	-	-	1	1	**6**	**6**	**5**
Aspidiotus nerii III	-	-	1	-	-	-	1	-	-	-	-	-	1	1	-	-	-	-	-	-	-	1	1	-	-	-	-	-	-	1	1	-	**8**	**8**	**6**
Chrysomphalus dictyospermi	-	-	-	-	-	-	-	-	-	-	-	-	-	-	-	1	-	-	-	-	-	-	-	-	-	-	-	-	-	-	-	1	**2**	**2**	**2**
Diaspidiotus ancylus	-	-	-	-	-	-	-	-	-	-	-	-	-	-	-	-	-	-	-	-	-	-	-	-	-	-	-	-	-	-	2	-	**2**	**1**	**1**
Diaspidiotus perniciosus	-	-	-	-	-	-	-	-	-	-	-	-	-	-	-	6	-	-	-	-	-	1	-	-	-	-	-	-	-	-	-	-	**7**	**2**	**2**
Diaspis chilensis	-	-	-	-	-	-	-	-	-	-	-	-	-	1	-	-	1	-	-	-	-	-	-	-	-	-	-	-	-	-	-	-	**2**	**2**	**2**
Diaspis echinocacti	-	-	-	-	-	-	-	-	-	-	-	-	-	-	-	-	-	-	-	-	-	-	-	-	-	-	1	-	-	-	-	-	**1**	**1**	**1**
Epidiaspis leperii	-	-	-	-	-	-	-	-	-	-	-	-	-	-	-	1	-	-	-	-	-	-	-	1	-	-	-	-	-	-	-	-	**2**	**2**	**2**
Furchadaspis zamiae	-	-	-	-	-	-	-	-	-	-	-	-	-	-	-	-	-	-	-	-	1	-	-	-	-	-	-	-	-	-	-	-	**1**	**1**	**1**
Hemiberlesia lataniae	-	-	1	-	1	-	-	-	-	-	-	-	-	-	1	-	-	-	-	-	1	-	-	-	-	-	-	-	1	-	-	1	**6**	**6**	**6**
Hemiberlesia palmae	-	-	-	-	-	-	-	-	-	-	-	-	-	-	-	-	-	-	-	-	-	-	-	-	-	-	-	-	-	-	1	-	**1**	**1**	**1**
Hemiberlesia rapax I	-	-	1	-	-	-	-	-	-	-	-	-	1	-	-	-	-	-	-	-	-	1	-	1	-	-	-	-	-	1	1	-	**6**	**6**	**6**
Hemiberlesia rapax II	-	-	-	-	-	-	-	1	-	-	-	-	1	-	-	-	-	-	-	-	2	-	-	1	-	-	-	-	-	-	1	-	**6**	**5**	**5**
Hemiberlesia rapax III	-	-	1	-	-	-	-	1	-	-	-	-	-	-	-	-	-	-	-	-	2	-	-	1	-	-	-	-	-	-	-	-	**5**	**4**	**4**
Lepidosaphes beckii	-	-	-	-	-	-	-	-	-	-	-	-	-	-	-	-	-	-	-	-	-	4	-	-	-	-	-	-	-	-	-	-	**4**	**1**	**1**
Lepidosaphes ulmi	-	-	1	-	-	-	-	-	-	-	-	-	1	-	-	2	-	1	-	-	-	-	-	-	-	-	-	-	-	-	-	-	**5**	**4**	**4**
Melanaspis sitreana	-	-	-	-	-	-	-	-	-	-	-	-	-	1	-	-	1	-	-	-	-	-	-	-	-	-	-	-	-	-	-	-	**2**	**2**	**2**
Pseudoparlatoria chilina	-	1	-	-	-	-	-	-	-	-	-	-	-	-	-	-	-	-	-	-	-	-	-	-	-	-	-	-	-	-	-	-	**1**	**1**	**1**
Pseudoparlatoria sp.	-	-	-	-	-	-	-	-	1	-	-	-	-	-	-	-	-	-	-	1	-	-	-	-	-	-	-	-	-	-	-	-	**2**	**2**	**2**
Ceroplastes sinensis	-	-	-	-	-	-	-	-	-	-	-	-	-	-	-	1	-	-	1	-	-	2	-	-	-	-	-	-	1	-	-	-	**5**	**4**	**4**
Ceroplastes sp. I	-	-	-	-	-	1	-	-	-	-	-	-	-	-	-	-	-	-	-	-	-	-	-	-	-	-	-	1	-	-	-	-	**2**	**2**	**2**
Ceroplastes sp. II	-	-	-	-	-	-	-	-	-	-	-	-	-	-	-	-	-	-	-	-	1	-	-	-	-	-	-	-	-	-	-	-	**1**	**1**	**1**
Coccidae unidentified	1	-	-	-	-	-	-	-	-	-	-	-	-	-	-	-	-	-	-	-	-	-	-	-	-	-	-	-	-	-	-	-	**1**	**1**	**1**
Coccus hesperidum	-	-	1	-	-	-	-	-	-	-	-	-	1	-	-	1	-	-	-	-	1	2	-	1	-	-	-	-	-	-	-	1	**8**	**7**	**6**
Parasaissetia nigra	1	-	-	-	-	-	-	-	-	-	-	-	-	-	-	-	-	-	-	-	-	-	-	-	-	-	-	-	-	-	-	-	**1**	**1**	**1**
Parthenolecanium corni	-	-	-	-	-	-	-	-	-	-	-	-	-	-	-	1	-	-	-	-	-	-	-	1	-	-	-	-	-	-	-	-	**2**	**2**	**2**
Protopulvinaria pyriformis	-	-	3	-	-	-	-	-	-	-	-	-	-	1	-	-	-	-	-	-	-	-	-	-	-	-	-	-	-	-	-	2	**6**	**3**	**3**
Pulvinariella mesembryanthemi	-	-	-	-	-	-	-	-	-	1	-	-	-	-	-	-	-	-	-	-	-	1	-	-	-	-	-	-	-	-	-	1	**3**	**3**	**3**
Saissetia coffeae	-	-	-	-	-	-	-	-	-	-	-	-	-	-	-	-	-	-	-	-	-	-	-	-	-	-	-	-	-	-	1	-	**1**	**1**	**1**
Saissetia oleae	-	-	2	-	-	-	-	-	-	-	1	1	1	1	-	5	-	-	-	-	2	6	1	-	1	1	-	-	1	1	2	-	**26**	**14**	**10**
